# METTL3 exerts synergistic effects on m6A methylation and histone modification to regulate the function of VGF in lung adenocarcinoma

**DOI:** 10.1186/s13148-023-01568-9

**Published:** 2023-09-23

**Authors:** Kesong Shi, Rula Sa, Le Dou, Yuan Wu, Zhiqiang Dong, Xinyao Fu, Haiquan Yu

**Affiliations:** State Key Laboratory of Reproductive Regulation a Breeding of Grassland Livestock, School of Life Sciences, Inner Mongolia University, Hohhot, 010070 Inner Mongolia China

**Keywords:** VGF, m6A modification, METTL3, Lung adenocarcinoma, Histone modification

## Abstract

**Background:**

Multiple genetic and epigenetic regulatory mechanisms play a vital role in tumorigenesis and development. Understanding the interplay between different epigenetic modifications and its contribution to transcriptional regulation in cancer is essential for precision medicine. Here, we aimed to investigate the interplay between N6-methyladenosine (m6A) modifications and histone modifications in lung adenocarcinoma (LUAD).

**Results:**

Based on the data from public databases, including chromatin property data (ATAC-seq, DNase-seq), methylated RNA immunoprecipitation sequencing (MeRIP-seq), and gene expression data (RNA-seq), a m6A-related differentially expressed gene nerve growth factor inducible (VGF) was identified between LUAD tissues and normal lung tissues. VGF was significantly highly expressed in LUAD tissues and cells, and was associated with a worse prognosis for LUAD, silencing of VGF inhibited the malignant phenotype of LUAD cells by inactivating the PI3K/AKT/mTOR pathway. Through the weighted correlation network analysis (WGCNA) and integration of TCGA-LUAD RNA-seq and m6A methyltransferase METTL3-knockdown RNA-seq data, a significant positive correlation between METTL3 and VGF was observed. By using the MeRIP-qPCR and dual-luciferase reporter assays, we demonstrated that METTL3 knockdown decreased m6A modification level of VGF coding sequences in LUAD cells, the colorimetric m6A quantification assay also showed that METTL3 knockdown significantly decreased global m6A modification level in LUAD cells. Interestingly, we found that METTL3 knockdown also reduced VGF expression by increasing H3K36me3 modification at the VGF promoter. Further research revealed that METTL3 knockdown upregulated the expression of histone methylase SETD2, the major H3K36me3 methyltransferase, by methylating the m6A site in the 3'UTR of SETD2 mRNA in LUAD cells.

**Conclusions:**

Overall, our results reveal that the expression of VGF in LUAD cells is regulated spatio-temporally by METTL3 through both transcriptional (via histone modifications) and post-transcriptional (via m6A modifications) mechanisms. The synergistic effect of these multiple epigenetic mechanisms provides new opportunities for the diagnosis and precision treatment of tumors.

**Supplementary Information:**

The online version contains supplementary material available at 10.1186/s13148-023-01568-9.

## Introduction

Lung cancer is one of the most common cancers and is divided into small cell lung cancer and non-small cell lung cancer (NSCLC) [1]. Lung adenocarcinoma (LUAD) is the most frequent histologic subtype of NSCLC and has high morbidity and mortality [2]. The overall prognosis for LUAD patients is poor. In recent years, epigenetics is having an increasingly important role in human diseases diagnosis and treatment [3].Therefore, further studies on epigenetic mechanism will be benefit to prevent the development of LUAD.

Epigenetic mechanisms of tumor cell growth are regulated at transcriptional, post-transcriptional, and translational levels [4, 5]. Various epigenetic mechanisms, such as DNA methylation and histone modifications, have been extensively studied and found to contribute to the development and progression of cancer [6]. Notably, the concerted action of different epigenetic modifiers is crucial for the precise spatiotemporal regulation of gene expression in cancer [7]. The “epitranscriptome” generated by chemical modification of internal RNA has drawn increasing attention on its role in many kinds of cancers [6, 8]. Among these modifications, N6-methyladenosine (m6A) RNA modification has emerged as a significant regulator of temporal and spatial gene expression during cancer development [9, 10]. The m6A modification is catalyzed by m6A RNA methyltransferases, and can be removed by the m6A RNA demethylases [11]. METTL3, the principal m6A methyltransferase, acts as the catalytic core [11] and has been shown to be involved in the dissemination and metastasis of lung cancer cells [12]. In addition, recent studies have highlighted the interplay between multiple epigenetic mechanisms in cancer development. For example, the removal of the histone mark H3K9me2 is specifically induced by m6A-modified transcripts [13]. Knockdown of METTL3 reduces the expression of histone methyltransferase EZH2 expression and global H3K27me3 levels via m6A modification during neurogenesis and neuronal development [14]. These multiple epigenetic mechanisms synergistically brought about some difficulties in diagnosis and treatment, but also provide novel avenues for cancer therapy. Therefore, investigating the synergy between METTL3-mediated m6A modifications and histone modifications in LUAD holds promise for precision therapy.

This study aims to investigate the role of METTL3 in LUAD, its regulatory impact on target molecules, and the synergistic effect of multiple epigenetic mechanisms. Additionally, we analyze the potential implications of METTL3 in LUAD diagnosis and treatment.

## Results

### Screening of key biomarkers in LUAD

**Fig. 1:**
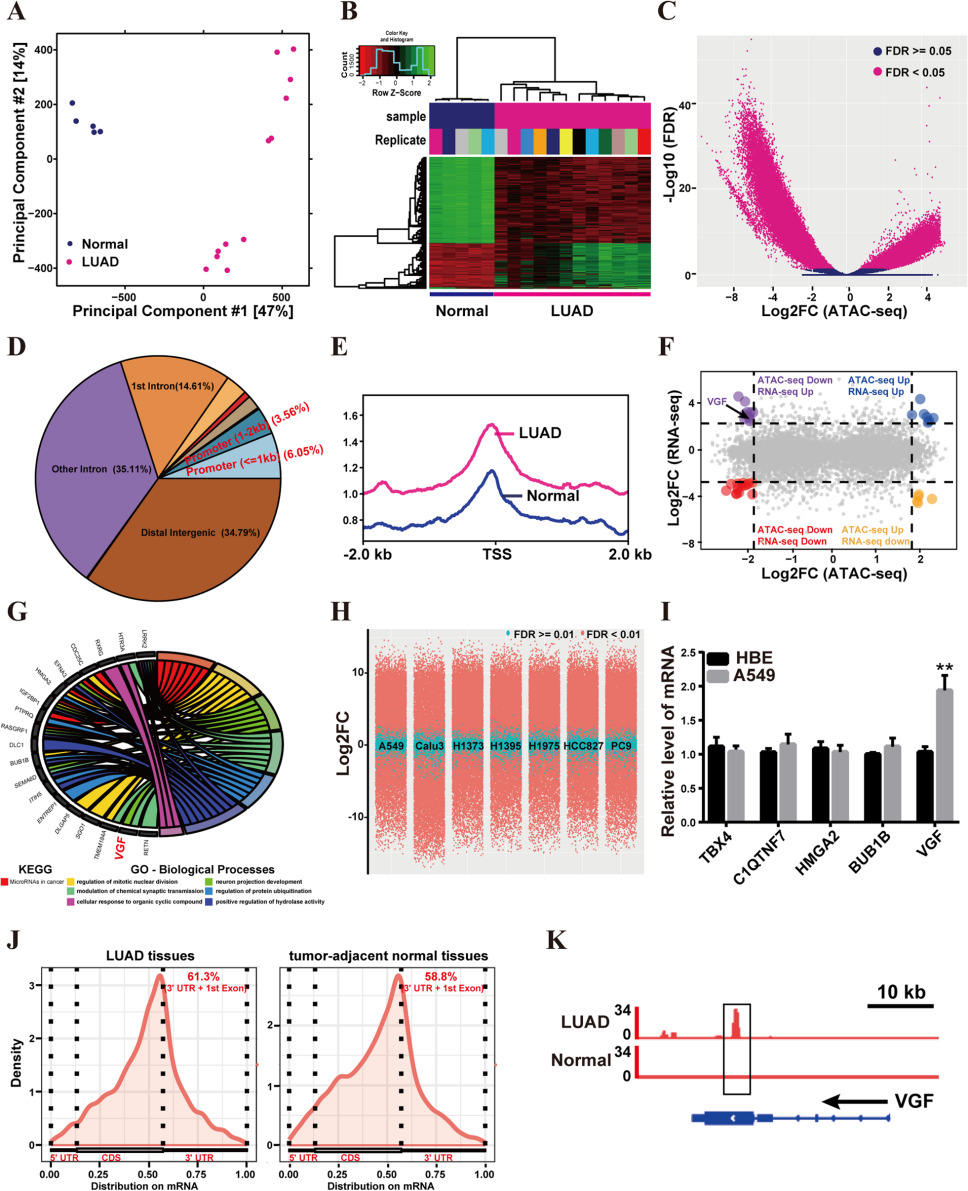
Screening of key biomarkers of LUAD. **A** Principal component analysis (PCA) analysis of the chromatin property data between LUAD tissues and normal lung tissues. **B** Heatmap showing affinities for differentially bound sites. **C** Volcano plot of differential peak expression analysis. Significantly differentially expressed peaks are denoted by red circles. **D** Distribution of promoter, exons, introns, and intergenic regions. **E** Line plot shows the chromatin accessibility in LUAD tissues and normal lung tissues around the TSS. **F** Volcano plot of differentially expressed genes. **G** Gene Ontology biological process and KEGG enrichment analysis. **H** Volcano plot for differential gene expression between LUAD cells and HBE cells. **I** Quantitative real-time polymerase chain reaction (qRT-PCR) verification of *TBX4*, *C1QTNF7*, *HMGA2*, *BUB1B*, and *VGF* gene expression. **J** mRNA density coverage. CDS: coding sequences. **K** Integrative Genomics Viewer (IGV) tracks displaying MeRIP-seq read distributions in VGF. Bar = mean ± standard deviation (SD). **p < 0.01

Given the complexity of gene expression regulation, it is essential to probe biological questions at different levels. Thus, multi-omics analysis is gaining importance. To search for key biomarkers of LUAD, chromatin property data (ATAC-seq, DNase-seq) and gene expression data (RNA-seq) were integrated. Initially, we conducted chromatin accessibility profiling analysis in LUAD tissues and normal lung tissues using publicly available data. Principal component analysis (PCA) (Fig. 1A) and hierarchical clustering analysis (Fig. 1B) revealed significant expression pattern differences between LUAD tissues and normal lung tissues. We observed a total of 18,114 differentially accessible peaks (fold change >|3.5|, false discovery rate FDR < 0.05) in LUAD tissues compared to normal lung tissues (Fig. 1C), and 9.61% of these differential peaks were located within the promoter region (the 2 kb region upstream and downstream) (Fig. 1D). Moreover, a large proportion of peaks were located close to the transcription start site (TSS) (Fig. 1E).

Next, through integrating the differentially expressed genes between LUAD samples and adjacent non-tumor samples (TCGA-LUAD RNA-seq), 57 overlapping differentially expressed genes were identified in both ATAC-seq and RNA-seq (Fig. 1F). Survival analysis revealed that 26 out of these 57 overlapping genes were associated with prognosis (Additional file 1: Table S1). To gain insight into the functional roles of these genes in LUAD, we performed Gene Ontology (GO) analysis and Kyoto Encyclopedia of Genes and Genomes (KEGG) enrichment analysis. GO analysis revealed that these genes were significantly associated with the regulation of mitotic nuclear division, neuron projection development, modulation of chemical synaptic transmission, regulation of protein ubiquitination, positive regulation of hydrolase activity, and cellular response to organic cyclic compound (Fig. 1G). KEGG enrichment analysis showed that these genes were mainly enriched in microRNAs in cancer (Fig. 1G). In addition, RNA-seq data from LUAD cells (A549, Calu3, H1373, H1395, H1975, HCC827, and PC9) and the human normal lung epithelial cell line HBE cells were analyzed (Fig. 1H). Among the 26 prognosis-associated genes, we found that five genes (*TBX4*, *C1QTNF7*, *HMGA2*, *BUB1B*, and *VGF*) exhibited differential expression (log2-fold change (*log2FC*) >|2.5|, adjusted *p* value < 0.01). Subsequent cellular experiments revealed that only VGF mRNA was highly expressed in A549 cells relative to HBE cells (Fig. 1I). Additionally, we reanalyzed the published MeRIP-seq data (GSE198288) of three pairs of LUAD samples and tumor-adjacent normal samples. Results showed that the highest number of m6A peaks in the stop codon and 3'untranslated regions (UTR) (Fig. 1J). Importantly, the m6A occupancy of VGF in LUAD tissues was increased compared to tumor-adjacent normal tissues (Fig. 1K).

### VGF knockdown repressed the malignant phenotype of LUAD cells by inactivating the PI3K/AKT/mTOR pathway

**Fig. 2:**
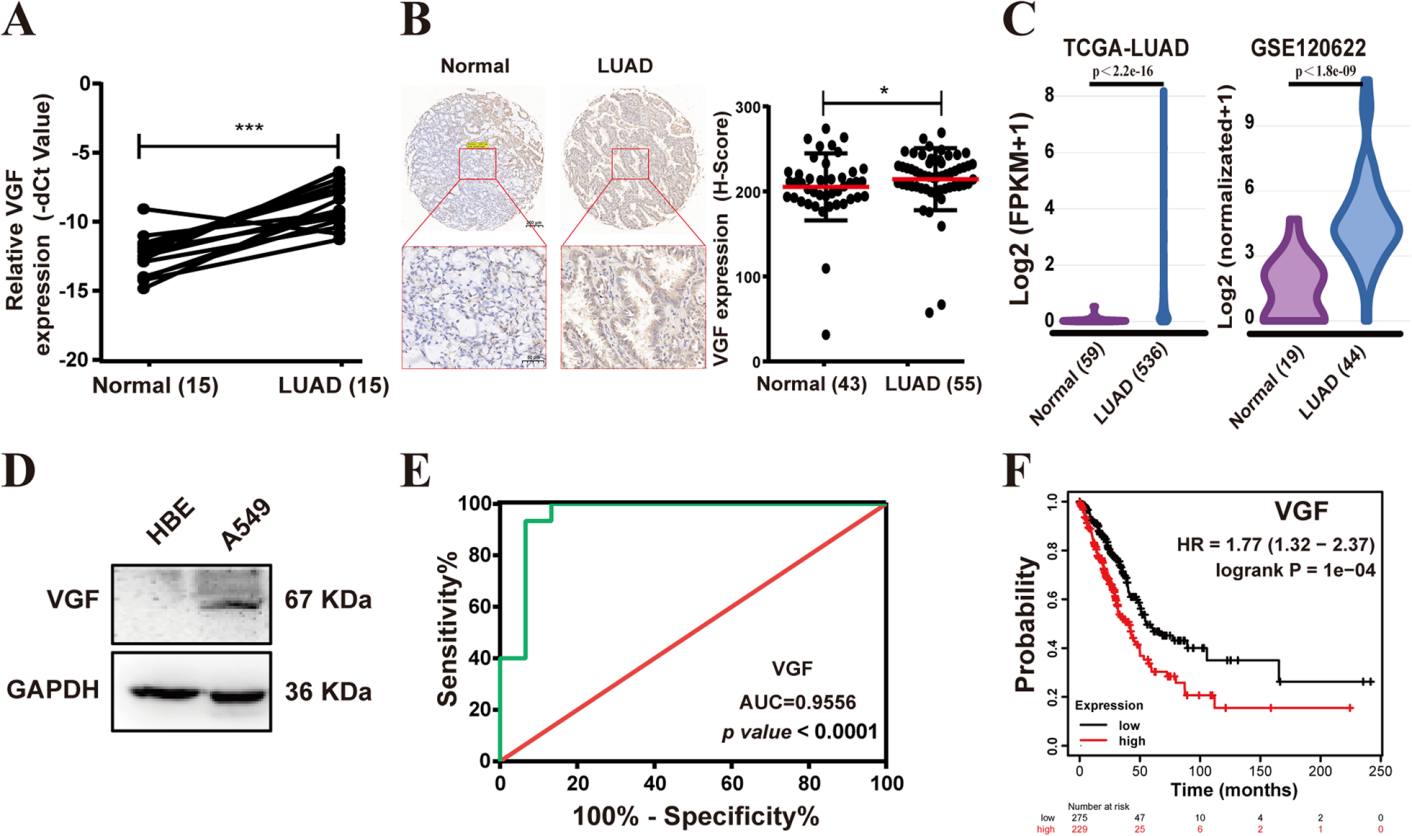
VGF is highly expressed in LUAD tissues and cells. **A** Validation VGF expression levels of cDNA microarray by qRT‒PCR. **B** Representative immunohistochemical staining for VGF in LUAD tissues and adjacent normal tissues (scale bar: 200 and 50 μm). **C** Relative VGF expression in LUAD tissues and adjacent normal tissues in TCGA and GSE120622. **D** Western blot measurement of VGF expression in A549 and HBE cells. **E** ROC curve analysis of the VGF gene. **F** Prognostic analyses for LUAD patients. Bar = mean ± SD. *p < 0.05, ***p < 0.001

To explore the potential role of VGF in LUAD, we examined its expression using qRT-PCR (Fig. 2A) and immunohistochemistry (Fig. 2B). The results demonstrated higher expression of VGF in LUAD tissues compared to adjacent normal tissues. Consistently, TCGA-LUAD and GSE120622 datasets also showed that VGF was highly expressed in LUAD tissues (Fig. 2C). This is consistent with the results of the LUAD cells (Figs. 1I, 2D). The receiver operating characteristic (ROC) curve was used to investigate the diagnostic value of VGF as a biomarker for LUAD. This result showed that the area under the ROC curve (AUC) was 0.9556 (Fig. 2E), suggesting that the expression profiles of VGF can distinguish LUAD tissues from adjacent normal tissues. Furthermore, low VGF expression was beneficial in the prognosis of LUAD patients (Fig. 2F).

**Fig. 3:**
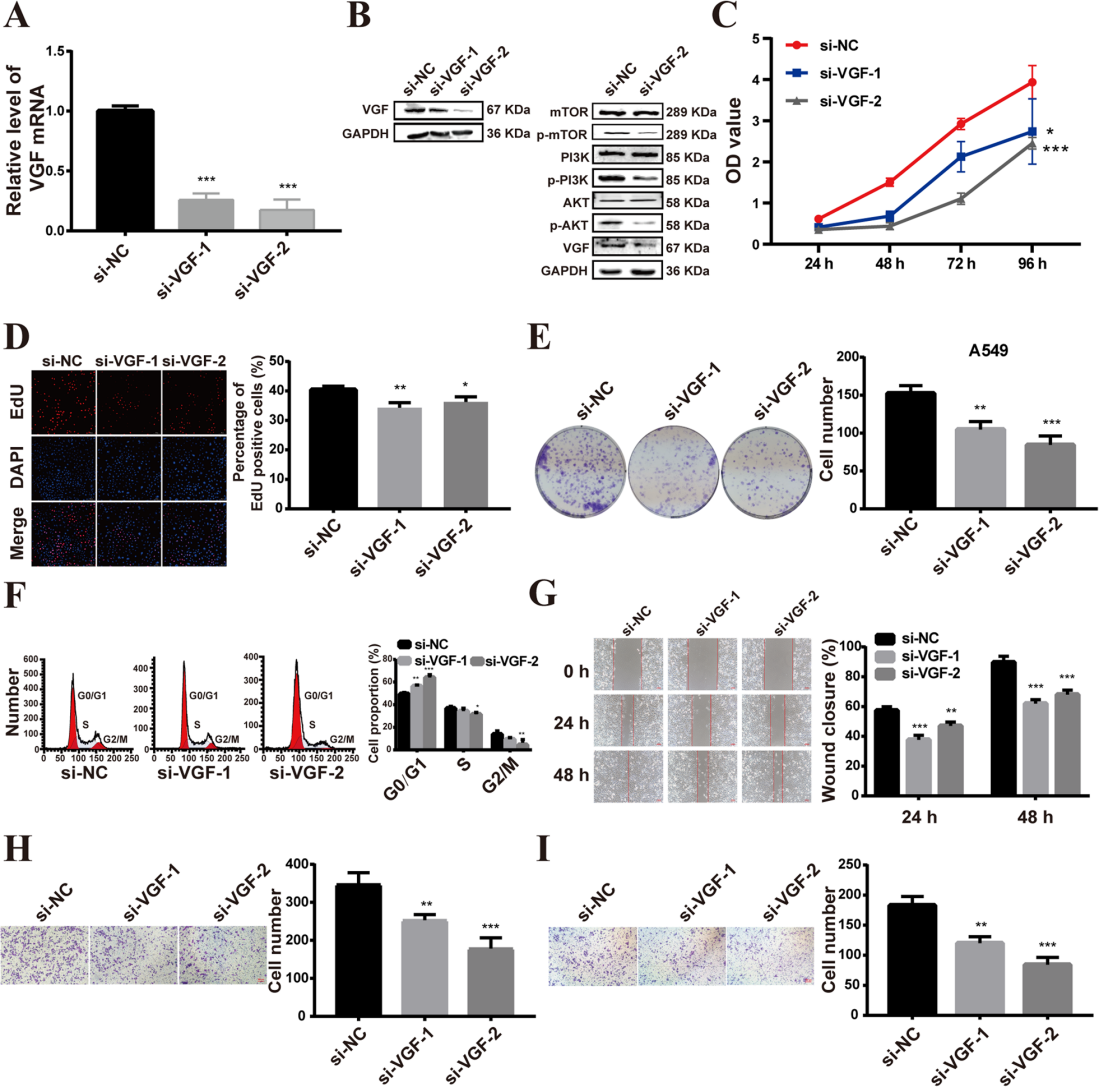
VGF knockdown repressed the malignant phenotype of LUAD cells in vitro. **A** The efficiency of VGF knockdown was verified by qRT‒PCR. **B** Western blot analysis of VGF, PI3K, phosphorylated PI3K (p-PI3K), AKT, p-AKT, p-mTOR and mTOR protein expression. **C-D** CCK8 (**C**) and EdU (**D**) assays were used to test the effect of VGF on LUAD cell viability. **E** Cell colony formation assays. **F** Flow cytometric analysis of the cell cycle. **G** Scratch assay experiments on A549 cells. **H-I** Cell migratory (**H**) and invasive (**I**) abilities were detected using transwell assays in A549 cells. Bar = mean ± SD. *p < 0.05, **p < 0.01, ***p < 0.001

To further explore the function of VGF in LUAD, we transfected VGF small interfering RNA siRNA (si-VGF) into A549 cells. Relative to the control si-NC, VGF expression was significantly reduced in A549 cells after si-VGF transfection (Fig. 3A, B). CCK8 and 5-ethynyl-2’-deoxyuridine (EdU) assays demonstrated that VGF knockdown significantly suppressed the viability of A549 cells (Fig. 3C, D). VGF knockdown also significantly inhibited the colony formation of A549 cells (Fig. 3E). Additionally, VGF knockdown increased the proportion of A549 cells in the G0/G1 phase (Fig. 3F). Wound healing (Fig. 3G) and transwell migration assay (Fig. 3H) revealed that VGF interference significantly inhibited migration of A549 cells. Transwell invasion assay (Fig. 3I) showed VGF knockdown reduced the invasion of A549 cells. Considering that PI3K/AKT/mTOR signalling pathway has been extensively reported as a pivotal network in regulating the lung cancer development [15, 16]. Therefore, we evaluated the effect of VGF on PI3K/AKT/mTOR signaling pathway. Western blot analysis showed that VGF knockdown decreased the phosphorylation levels of PI3K, AKT and mTOR (Fig. 3B). These results indicate that VGF knockdown represses the malignant phenotypes of LUAD cells by inactivating the PI3K/AKT/ mTOR pathway.

### Screening of m6A regulators associated with VGF

**Fig. 4:**
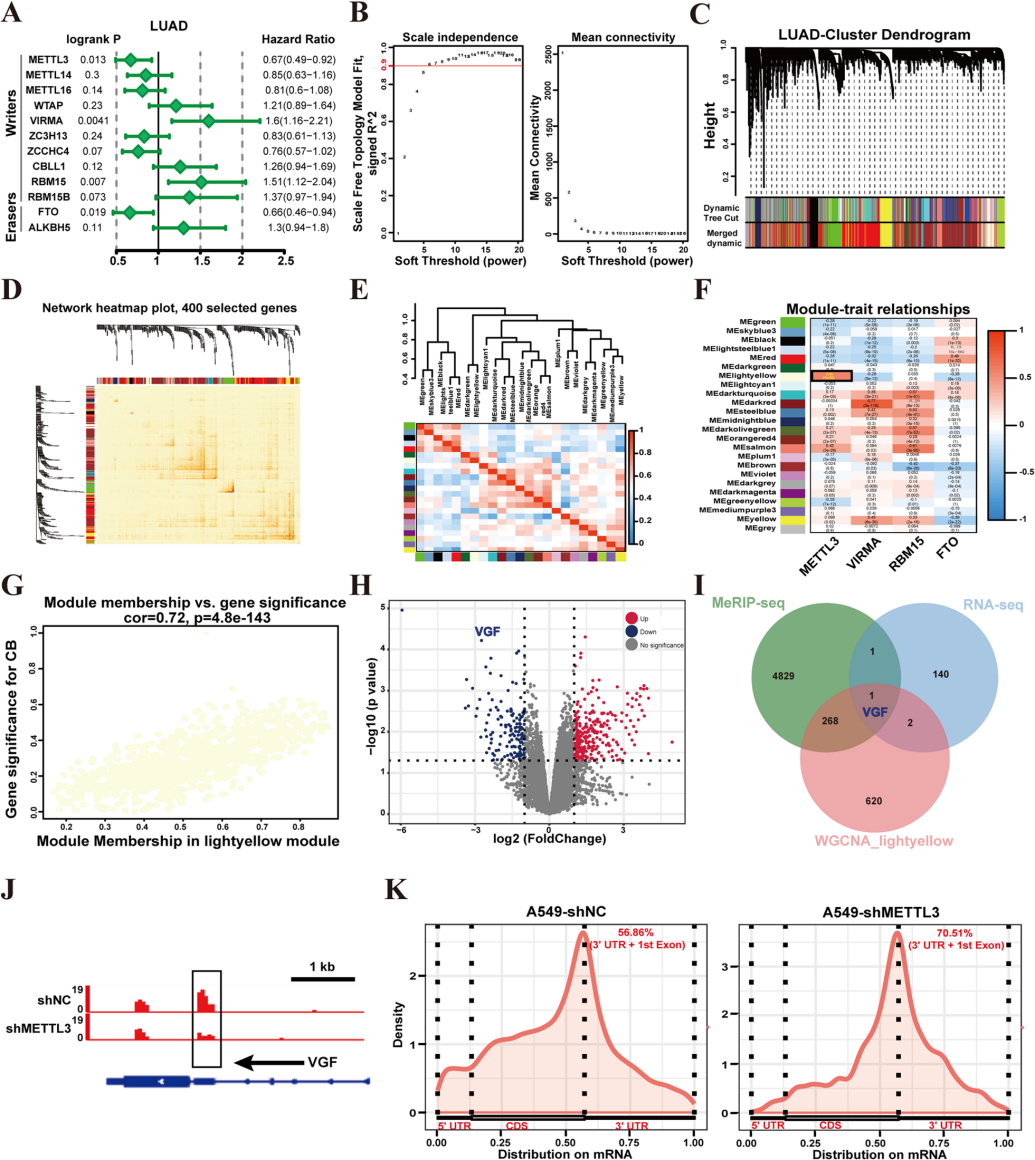
Screening of m6A regulators associated with VGF. **A** Forest map illustrates the survival analysis of m6A methylases and demethylases in LUAD. **B** The scale-free topology fitting index R^2^ reaches 0.9. **C** A hierarchical clustering tree is shown in LUAD. **D** Interaction relationship analysis of co-expressed genes. **E** The network heatmap visualized the interactions between the 24 modules. **F** Correlation between the gene module and m6A-related genes in LUAD. **G** The light-yellow module was significantly positively correlated with the module genes. **H** Volcano plot of differential gene expression in METTL3-knockdown A549 cells compared to control cells. **I** Venn diagrams were used to screen the VGF gene. **J** Integrative Genomics Viewer (IGV) tracks displaying MeRIP-seq read distributions in VGF. **K** mRNA density coverage

N6-methyladenosine (m6A) is an abundant reversible post-transcriptional modification and plays a crucial role in the malignant phenotype of tumor cells [17, 18]. Previous studies have reported associations between m6A and lung cancer malignancy [18, 19]. Our previous study found increased m6A occupancy of VGF in LUAD tissues compared to adjacent normal tissues (Fig. 1K). This finding led us to speculate that VGF expression is dependent on m6A modification. To verify this speculation, we initially analyzed the association between m6A regulators (methyltransferases and demethylases) and the prognosis of LUAD. The results showed that the METTL3, VIRMA, RBM15, and FTO expression were found to be significantly associated with LUAD prognosis (logrank *p* < 0.05) (Fig. 4A).

Next, we performed WGCNA analysis using the mRNA expression matrix and the identified m6A regulators. The relatively balanced scale independence and average connectivity of WGCNA were identified. The scale-free topology fitting index R^2^ reaches 0.9 (Fig. 4B), and a hierarchical clustering tree was obtained using the dynamic cutting method (Fig. 4C). Moreover, interaction relationship analysis of co-expression genes revealed high independence in relative gene expression within each module. A network heatmap visualized the interactions between the 24 modules, confirming their independence (Fig. 4D, E). Subsequently, the m6A-related module was screened (correlation coefficient (*R*) > 0.5, *p* value < 0.01). Among these modules, the light-yellow module showed a significant positive correlation (*R* = 0.56, *p* value = 1e−49) with METTL3, and contained the VGF gene (Fig. 4F). Additionally, the correlation between module membership (MM) and gene significance (GS) was significant in the light-yellow module (*R* = 0.72, *p* value = 4.8e−143) (Fig. 4G).

To further support our findings, we reanalyzed the published METTL3-knockdown RNA-seq data from A549 cells (GSE76367). The analysis revealed significantly downregulated expression levels of VGF mRNA (*log2FC* > 2.5, *p* value < 0.01) in A549 cells following METTL3 knockdown (Fig. 4H). Furthermore, published MeRIP-seq data from A549 cells (GSE76367) provided 9298 m6A peaks (in 5099 genes), including VGF, as identified by Lin et al. [18]. By comparing the METTL3-knockdown RNA-seq, MeRIP-seq data, and the genes of the light-yellow module, we found a single overlapping gene VGF (Fig. 4I). Further analysis found decreased m6A occupancy of VGF in METTL3-depleted A549 cells compared to control cells from the MeRIP-seq data (GSE55572) (Fig. 4J), and the largest number of m6A peaks was found in the stop codon and 3'UTR of both METTL3-depleted A549 cells and control cells (Fig. 4K). These results suggest that VGF expression could be positively regulated by METTL3-mediated m6A modification.

### METTL3 regulates the expression of VGF via m6A modification

**Fig. 5:**
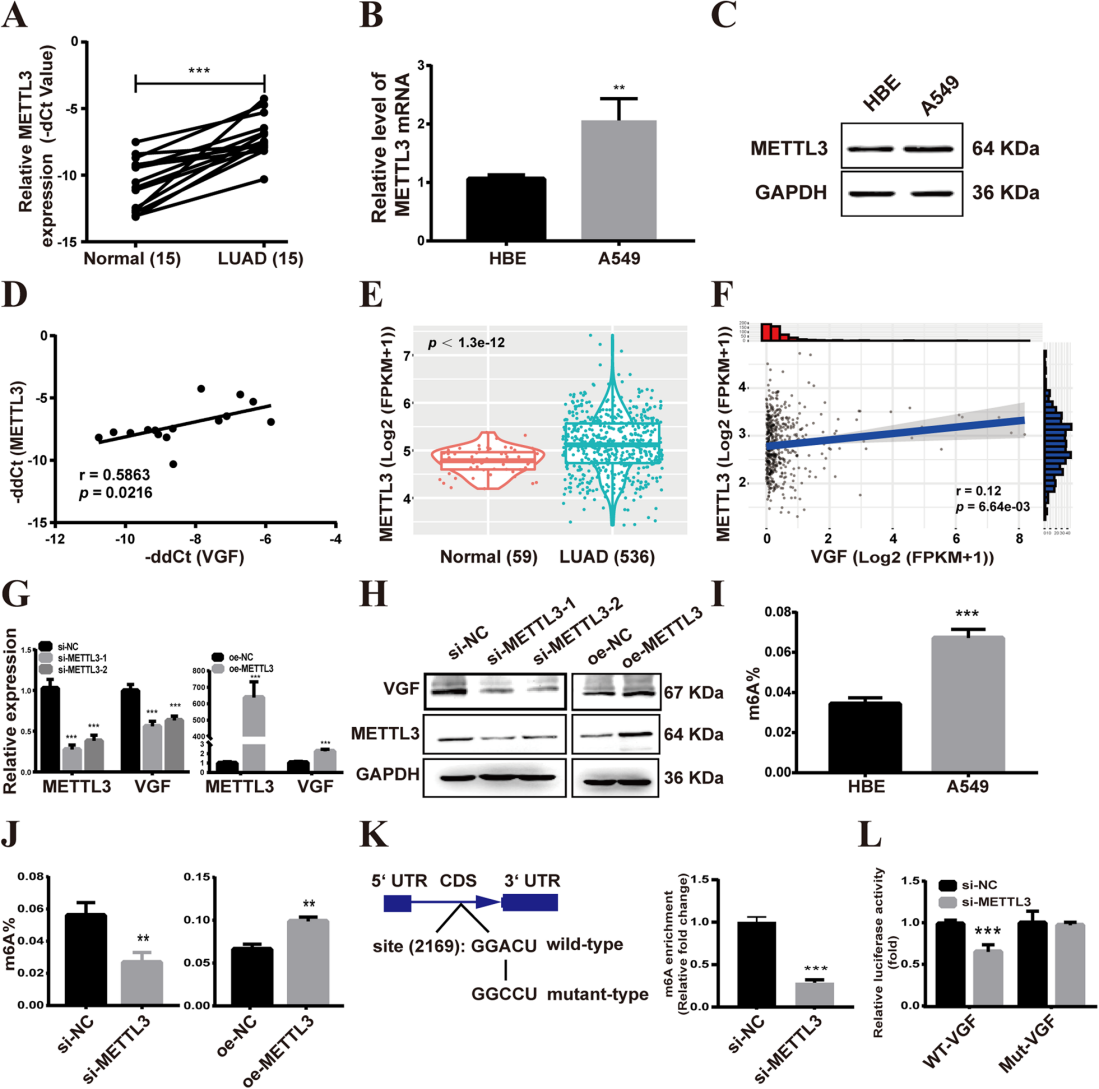
METTL3 regulates the expression of VGF via m6A modification. **A** Validation of METTL3 expression levels in the cDNA microarray by qRT‒PCR. **B**, **C** qRT‒PCR (**B**) and western blot (**C**) measurement of METTL3 expression in A549 and HBE cells. **D** Correlations between the expression of METTL3 and VGF in LUAD tissues. **E** Relative VGF expression in LUAD and adjacent noncancerous tissues in the TCGA. **F** Correlations between the expression of METTL3 and VGF in TCGA-LUAD. **G**, **H** The expression of METTL3 and VGF was verified by qRT‒PCR (**G**) and western blot (**H**). **I** The total m6A level of A549 and HBE cells. **J** The total m6A level of A549 cells after METTL3 overexpression and knockdown.** K** Prediction results of VGF mRNA in the SRAMP website and schematic photo of CDS-WT and CDS-mutant in VGF mRNA (left), VGF m6A modification levels in A549 cells determined using MeRIP-qPCR (right). **L** Luciferase activity was measured using a dual luciferase assay. Bar = mean ± SD. *p < 0.05, **p < 0.01, ***p < 0.001

In order to demonstrate the above speculation, we investigated the gene expression correlation between METTL3 and VGF. The results showed that METTL3 was highly expressed in LUAD tissues and cells (Fig. 5A–C) and was significantly positively correlated with VGF in LUAD tissues (Fig. 5D). This correlation was also observed in the TCGA-LUAD database (Fig. 5E, F). Furthermore, METTL3 and VGF levels in the METTL3 knockdown group were significantly decreased in comparison to the control group in A549 cells, whereas METTL3 overexpression had the opposite results (Fig. 5G, H). Next, the m6A level of total RNA in HBE and A549 cells was detected. Compared to the HBE cells, the m6A level was significantly increased in A549 cells (Fig. 5I), and METTL3 knockdown repressed the m6A content level, while METTL3 overexpression increased the m6A content (Fig. 5J). Using the SRAMP online tool [20], we identified a remarkable m6A site in the coding sequences (CDS) of VGF mRNA in A549 cells (Fig. 5K). Subsequently, MeRIP-qPCR and dual-luciferase reporter assays were performed to further verify the results. MeRIP-qPCR demonstrated that METTL3 knockdown significantly decreased the m6A levels of fragments related to the site (Fig. 5K). Additionally, the dual-luciferase reporter assays showed that METTL3 knockdown decreased the activity of luciferase with wild-type (WT) VGF but not mutated VGF (Fig. 5L). These findings suggest that METTL3 regulates VGF expression by methylating the m6A site in the CDS of VGF mRNA in LUAD cells.

### METTL3-mediated m6A modification of VGF mRNA promotes LUAD progression

**Fig. 6:**
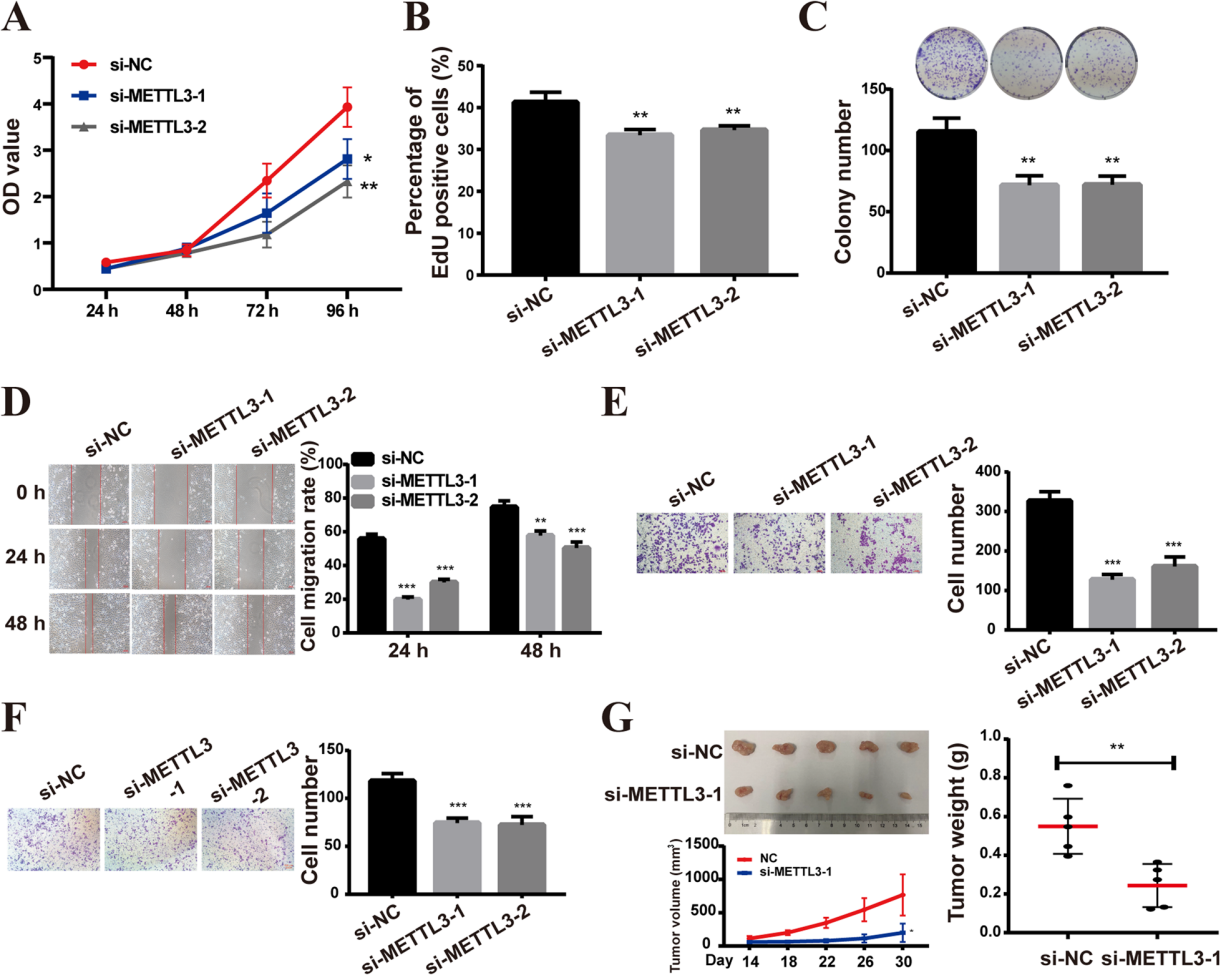
METTL3 knockdown inhibited the malignant phenotype of A549 cells in vitro. **A**, **B** CCK8 (**A**) and EdU (**B**) assays were used to assess cell viability and proliferation. **C** Colony formation assays. **D** The scratch assay experiments on A549 cells. **E**, **F** Cell migratory and invasive abilities were detected using transwell assays in A549 cells. **G** METTL3 knockdown inhibited the growth of subcutaneous xenografts in vivo (top left), the tumor growth curve (low left), and the weight of tumors xenografted in nude mice (right). Bar = mean ± SD. *p < 0.05, **p < 0.01, ***p < 0.001

Based on the previous results, it is necessary to further investigate whether VGF is regulated by METTL3 through m6A modification to promote the malignant phenotype of LUAD. Functional analyses, including cell proliferation, colony formation, cell migration, and invasion assays, were conducted to assess the effects of METTL3 knockdown on A549 cells in vitro. The results demonstrated that METTL3 knockdown inhibited the malignant phenotype of A549 cells (Fig. 6A–F), while overexpression of METTL3 had the opposite results (Additional file 2: Fig. S1A–S1F). Furthermore, in vivo experiments demonstrated that the METTL3 knockdown groups exhibited smaller tumors with slower growth compared to the control group (Fig. 6G).

**Fig. 7:**
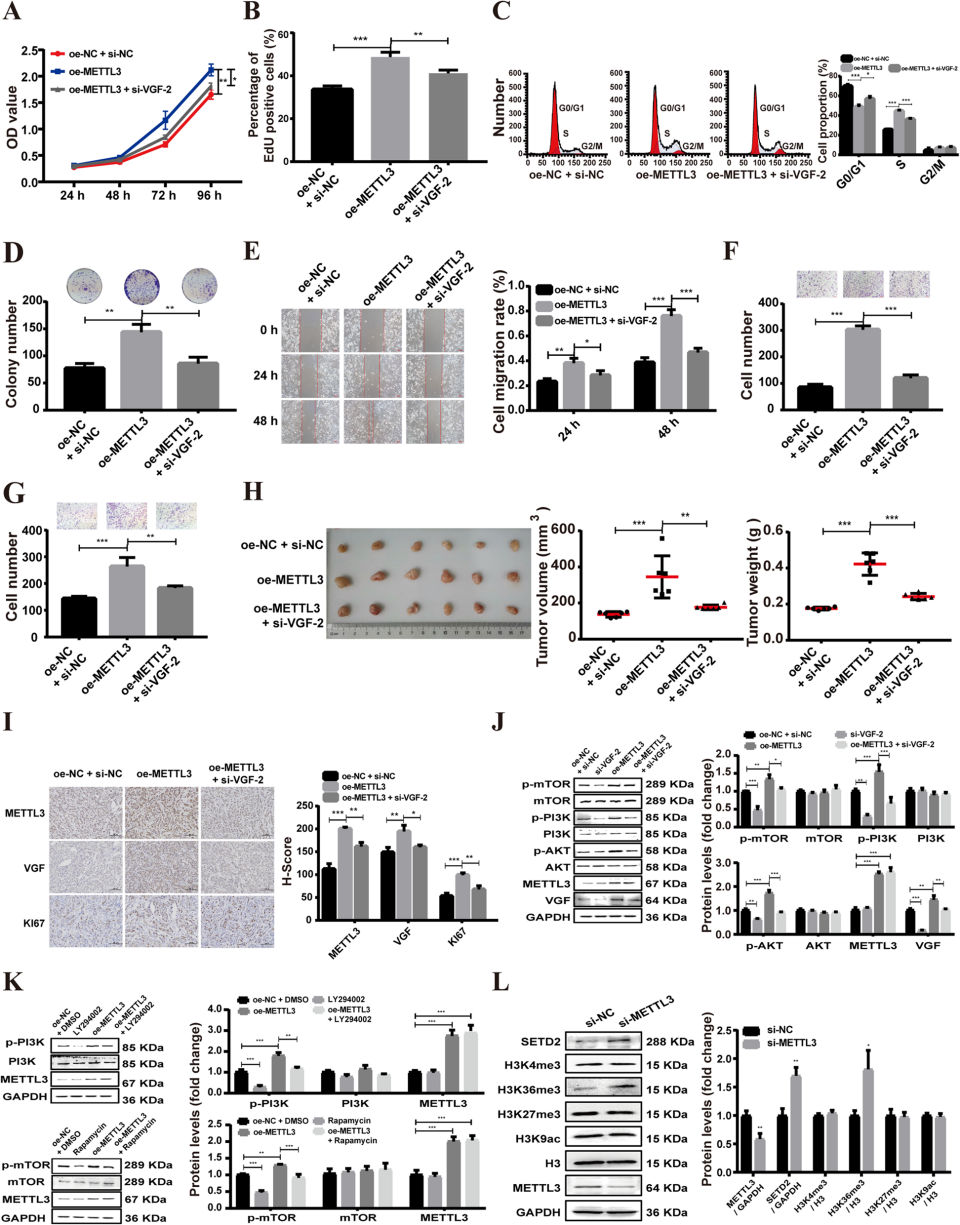
METTL3-mediated m6A modification of VGF mRNA promotes LUAD progression. **A**, **B** CCK8 (**A**) and EdU (**B**) assays were performed to assess cell viability and proliferation. **C** Flow cytometric analysis of the cell cycle. **D** Colony formation assays. **E** Scratch assay experiments on A549 cells. **F**, **G** Cell migratory and invasive abilities in A549 cells. **H** Gross appearance (left), tumor volume (middle), and tumor weight (right) of each group. **I** IHC staining results (METTL3, VGF, Ki-67). **J** Western blot results for METTL3, VGF, p-PI3K, PI3K, p-AKT, AKT, mTOR and p-mTOR. **K** Western blot was used to detect the effects of LY294002 and Rapamycin on the protein expression of p-PI3K and p-mTOR in A549 cells, respectively. **L** Western blot results of METTL3, SETD2, and H3 proteins

To investigate the role of VGF in METTL3-mediated oncogenic effects. siRNA targeting VGF was transfected into METTL3-overexpressing A549 cells. The results showed that VGF knockdown largely inhibited the promoting effect of METTL3 overexpression on A549 cell proliferation, colony formation, migration, and invasion (Fig. 7A–G). Moreover, in vivo experiments demonstrated that mice in the METTL3 overexpression group showed an obviously larger tumor size and tumor weight than the control group, while VGF knockdown inhibited METTL3 overexpression-induced tumor progression (Fig. 7H). Additionally, IHC staining showed that overexpression of METTL3 increased the expression of VGF and KI67, which was recovered by knockdown of VGF (Fig. 7I). Western blot analysis further indicated that overexpression of METTL3 increased the phosphorylation levels of PI3K, AKT and mTOR, which were attenuated by VGF knockdown (Fig. 7J), Moreover, the increases of the p-PI3K and p-mTOR expression induced by METTL3 overexpression was reversed by PI3K inhibitor LY294002 and mTOR inhibitor Rapamycin, respectively (Fig. 7K). The results suggest that METTL3 promotes LUAD progression by upregulating VGF and subsequently activating the PI3K/AKT/mTOR signaling pathway.

### METTL3 modulates the H3K36me3 level by mediating m6A modification of SETD2 mRNA

**Fig. 8:**
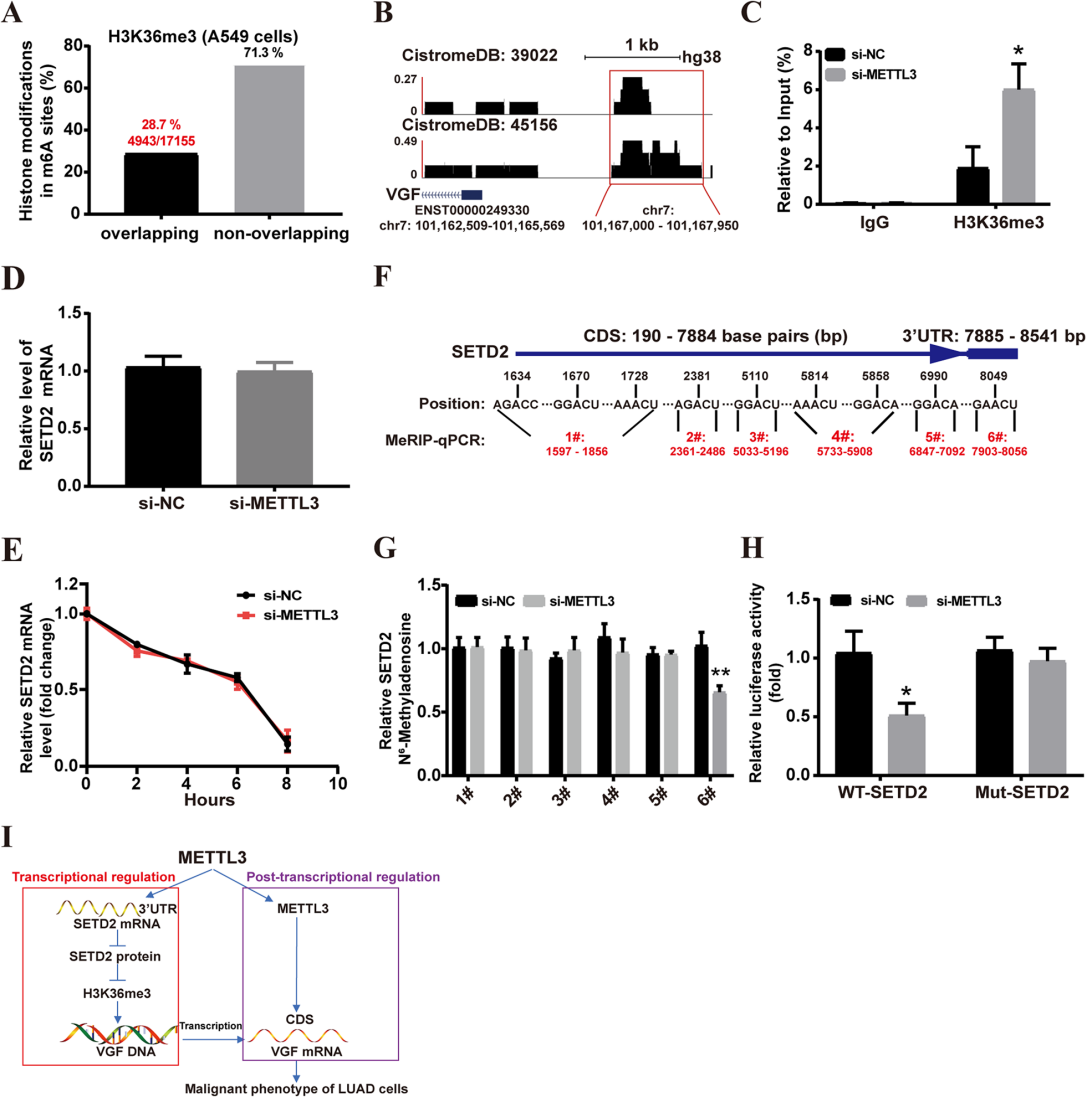
METTL3 modulates the H3K36me3 level by mediating m6A modification of SETD2 mRNA. **A** Overlaps of m6A sites with histone modification sites in A549 cells. **B** VGF enrichment around H3K36me3 ChIP-seq peak centers in A549 cells. **C** ChIP‒qPCR analysis of H3K36me3 at VGF. **D** The expression of SETD2 mRNA was verified by qRT‒PCR. **E** VGF mRNA was analyzed in A549 cells after actinomycin D treatment. **F** Predicted m6A sites in the CDS and 3'UTR sequence of SETD2 mRNA. **G** The m6A levels of m6A sites of SETD2 mRNA in A549 cells were analyzed by MeRIP-qPCR. **H** Luciferase activity was measured using a dual luciferase assay. **I** Schematic showing the functional and molecular mechanisms of METTL3 in LUAD. Bar = mean ± SD. *p < 0.05, **p < 0.01

The differences in the chromatin features of VGF between the LUAD tissues and normal lung tissues (Fig. 1F) indicate the involvement of chromatin organization in the control of VGF gene expression. Histone modifications, particularly Histone 3 (H3) proteins, play an important role in chromatin remodeling and gene expression [21]. Thus, we evaluated whether METTL3 regulates tri-methylation and acetylation of H3. The results showed that METTL3 knockdown led to increased abundance of H3K36me3, but not other H3 proteins, compared to the control (Fig. 7L). Furthermore, by comparing the distribution of m6A in A549 cells to that of H3K36me3 histone modifications, we found that 28.7% of m6A peaks overlapped with the H3K36me3 modification (Fig. 8A). Additionally, an enrichment of H3K36me3 was observed at the upstream region of the transcription start site (TSS) of VGF genes in A549 cells (Fig. 8B). ChIP‒qPCR data demonstrated that METTL3 knockdown increased H3K36me3 levels at the promoter region of VGF in A549 cells (Fig. 8C).

To further investigate how METTL3 regulates the H3K36me3 histone mark. Based on METTL3-knockdown RNA-seq in A549 cells, we analyzed the total RNA levels of histone methylase SETD2 (the major H3K36me3 methyltransferase) after METTL3 knockdown. The results showed no significant change was observed in the RNA levels of SETD2 (*log2FC* = 0.068, *p* value = 0.55) compared with controls (Fig. 4H). Consistent with METTL3-knockdown RNA-seq results, qRT‒PCR revealed that METTL3 knockdown did not impact the expression of SETD2 mRNA in A549 cells (Fig. 8D). Further analysis demonstrated that the stability of SETD2 mRNA, in response to actinomycin D treatment, exhibited no significant difference between METTL3 knockdown and control group (Fig. 8E). However, SETD2 protein expression was significantly upregulated in METTL3-knockdown A549 cells compared to controls cells (Fig. 7L). Additionally, the SRAMP online tool identified nine potential m6A sites with high confidence in the CDS and 3'UTR of SETD2 mRNA in A549 cells (Fig. 8F). To validate these predicted m6A sites, specific primers were designed to amplify nine sites, which were combined into one region of less than 200 base pairs (bp). The MeRIP-qPCR assay and dual-luciferase reporter assay was conducted to determine the enrichment of m6A in SETD2. MeRIP-qPCR showed that only one m6A site (position 6#) was significantly decreased in the SETD2 3'UTR in METTL3-knockdown A549 cells (Fig. 8G). The dual-luciferase reporter assays found that METTL3 knockdown significantly declined the relative luciferase activity of SETD2 3ʹUTR-WT, but the relative luciferase activity of SETD2 3ʹUTR-Mut was not affected (Fig. 8H). These results suggest that METTL3 regulates SETD2 translation by methylating the m6A site in the 3'UTR of SETD2 mRNA in LUAD cells.

## Discussion

Lung adenocarcinoma (LUAD) is the most common histologic subtype of NSCLC and is associated with high morbidity and mortality rates [2]. Currently, the overall survival rate for LUAD patients is poor, highlighting the need for further exploration of underlying mechanisms and identification of potential biomarkers. In this study, based on chromatin property data (ATAC-seq, DNase-seq) and gene expression data (RNA-seq), overlapping differentially expressed gene were screened between LUAD tissues and normal lung tissues, and m6A-related overlapping differentially expressed gene VGF was subsequently found by using m6A MeRIP‐seq data, suggesting an important role for the VGF in LUAD. VGF was originally identified in the neurons and neuroendocrine cells and is responsible for energy balance and metabolism [22]. Apart from depression, Alzheimer’s disease (AD), and other neuroendocrine diseases, VGF has been associated with cancer [23]. Previous studies have shown that VGF promotes the malignant phenotype of pancreatic neuroendocrine tumors [24]. Moreover, VGF is highly expressed in LUAD tissues and cells, and its high expression predicts poor prognosis [25]. However, the detailed mechanism of VGF in LUAD has been poorly studied. In our study, we found that VGF promotes the malignant phenotype of LUAD cells by activating the PI3K/AKT/mTOR pathway, suggesting that VGF could serve as a potential biomarker and therapeutic target for LUAD.

Epigenetic mechanisms of gene expression in cancer development are regulated at transcriptional, post-transcriptional, and translational levels [4, 5]. In recent years, the regulatory roles of post-transcriptional modulations, especially N6-methyladenosine (m6A) RNA modification, in controlling gene expression in lung cancer have gained increasing attention [18, 26]. However, research on the regulation of VGF by m6A modification has been limited to bioinformatics analysis [27]. Ding et al. found that VGF may be regulated by m6A mRNA demethylation FTO through the RNA-seq analysis of FTO-overexpressing A549 cells and control A594 cells [27]. Here, we discovered that METTL3 promote the malignant phenotypes of LUAD both in vitro and in vivo. In addition, we identified a significant m6A site in the CDS of VGF mRNA in A549 cells, and further research found that METTL3 knockdown reduced the expression of VGF by methylating the m6A site in the CDS of VGF mRNA in LUAD cells. Previous studies on VGF regulation have primarily focused on genetic mechanisms [28, 29]. Therefore, our results significantly enhance our understanding of the precise regulation of the VGF expression in cancer.

Interestingly, we found that METTL3 knockdown not only decreased the expression of VGF via m6A modification but also inhibited VGF expression by increasing H3K36me3 modification at the VGF promoter. Similar to m6A modification, histone modifications are among the epigenetic mechanisms that control gene expression [30]. Recent studies have unveiled a crosstalk between m6A modification and histone modification. For example, H3K4me3 (in erythroid progenitors) [31] and H3K36me3 (in embryonic stem cells) [32] have been associated with METTL3-mediated m6A modifications. Taken together with our results, these earlier studies suggest an association between METTL3-mediated m6A modifications and histone modification in different biological processes. In addition, it has been demonstrated that m6A marks on chromosome-associated regulatory RNAs and retrotransposon transcripts regulate chromatin modification and transcription state in mouse ESCs by recruiting m6A reader YTHDC1 [33, 34]. However, we observed a significant association between the expression of various other m6A reader proteins (YTHDF1, YTHDF2, YTHDC2, IGF2BP1, IGF2BP2, IGF2BP3, HNRNPC, and HNRNPA2B1), except YTHDC1, and the prognosis of LUAD patients (Additional file 2: Fig. S1G). Further research is needed to elucidate the role of m6A readers in the interplay between m6A modifications and histone modifications in LUAD.

Notably, SETD2 is the main methyltransferase that catalyzes the formation of H3K36me3 [35]. Therefore, we examined SETD2 expression in METTL3 knockdown A549 cells, results found that METTL3 knockdown significantly increased SETD2 protein expression. Further investigation revealed that the upregulation of SETD2 in METTL3 knockdown A549 cells was due to decreased the m6A levels in the 3'UTR of SETD2 mRNA. This finding suggests that METTL3 modulates the global H3K36me3 levels by mediating m6A modification of SETD2 mRNA in LUAD cells. Recently, inhibitors of the epigenetic regulator METTL3 have emerged as potential cancer therapies and are currently under evaluation in clinical trials [36, 37]. Thus, our study provided a new mechanistic vista for epigenetic-directed therapies of LUAD.

There is a limitation to this study. We did not evaluate the study in multiple lung adenocarcinoma cell lines, which may affect the generalizability of our findings.

## Conclusions

In conclusion, our study highlights the underlying molecular mechanism of the VGF pro-oncogenic effects. Further research found that METTL3 promotes the malignant phenotype of LUAD cells through transcriptional (histone modification) and post-transcriptional modification (m6A) of VGF (Fig. 8I), revealing a crosstalk between histone modification and m6A methylation. Understanding of specific regulation mechanism of METTL3 will facilitate the development of future epigenetics biomarkers and precision therapies.

## Materials and methods

### Data sources

The chromatin property data of five Homo sapiens lung tissues were derived from the GSE18927 dataset. Chromatin property data of 14 LUAD tissue samples were collected from the study by Azumi et al. [38]. MeRIP-seq data of three pairs of LUAD samples and tumor-adjacent normal tissues were derived from GSE198288 dataset, and METTL3-knockdown RNA-seq and MeRIP-seq in A549 cells were collected from GSE76367 and GSE55572 datasets, respectively. In addition, RNA-seq data of LUAD tissue samples were collected from TCGA-LUAD and GSE120622 dataset. The RNA-seq data of LUAD cell lines (A549, Calu3, H1373, H1395, H1975, HCC827, and PC9) and human bronchial epithelial cells HBE were derived from GSE160683 and GSE85402 datasets, respectively. H3K36me3 ChIP-seq data in the A549 cells was derived from the Cistrome Data Browser (http://www.cisttrome.org/db).

### Bioinformatics analysis

ATAC-seq, DNase-seq, RNA-seq, and MeRIP-seq data were obtained as described above. Our previous studies described the detailed data processing procedures for ATAC-seq, DNase-seq, and RNA-seq [39]. Briefly, raw sequences for ATAC-seq, DNase-seq, and RNA-seq data were processed by Cutadapt v1.9. Subsequently, the reads were aligned to the reference genome (hg38) using Bowtie2 v2.4.4 [40] or HISAT2 v2.2.1 [41]. Moreover, peaks of ATAC-seq and DNase-seq were called by using MACS2 v2.2.7.1 [42], and differential peak analysis was performed using DiffBind v3.4.0, while differential gene expression analysis of RNA-seq was assessed using limma v3.48.3. For MeRIP-seq data, raw reads were filtered by Cutadapt v1.9, and the rRNA was digitally removed using Bowtie2 v2.4.4. Then, the data were aligned to the reference genome (GRCh38) using HISAT2 v2.2.1. Peak calling and differential peak analysis were conducted using exomePeak2 v1.4.2 [43], and the positions of the m6A peaks were determined and visualized using the Guitar (v2.8.0) package [44]. Furthermore, the functional annotation and pathway enrichment of the differentially expressed genes was conducted using DAVID (*p* value < 0.05) [45]. For WGCNA, a power of β = 6 and a scale-free R^2^ = 0.90 were selected as soft-threshold parameters. The prognostic values of m6A regulators and VGF at the mRNA level were determined using the Kaplan‒Meier plotter (http://kmplot.com/analysis/).

### Tumor samples

The cDNA microarray of LUAD tissues (HLugA030PG01) was purchased from Shanghai Outdo Biotech Company (Shanghai, China), which contains 15 LUAD tissues and 15 adjacent noncancerous tissues. Additionally, 55 LUAD tissues and 43 adjacent noncancerous tissues (HLugA098Bc01) were obtained from the same company (Shanghai Outdo Biotech Company). The study involving human participants was approved by the Ethics Committee of Shanghai Outdo Biotech Company with NO. YB M-05-02.

### Cell culture, gene overexpression and knockdown

The human LUAD cell line A549 and human normal lung epithelial cell line HBE (American Type Culture Collection, Manassas, VA, USA) were cultured in DMEM (VivaCell, Shanghai, China) containing 10% fetal bovine serum. For overexpression of METTL3 in A549 cells, the coding sequences (CDS) full-length sequence of human METTL3 mRNA was amplified and inserted into the pIRES2-ZsGreen1 vector to construct the overexpression plasmid. For knockdown of METTL3 or VGF in A549 cells, small interfering RNA (siRNA) targeting METTL3 or VGF was designed and synthesized by the Genepharma (Shanghai, China). The sequences of the siRNAs are provided in Additional file 1: Table S2. The aforementioned METTL3 siRNA and VGF siRNA and their controls were individually transduced into A549 cells using JetPRIME reagent (Polyplus-transfection S.A., Illkirch-Graffenstaden, France). In addition, to investigate the PI3K/AKT/mTOR pathway in A549 cells, which were treated with Rapamycin (100 nM, HY-10219, MCE), the PI3K inhibitor LY294002 (20 μM, HY-10108, MCE) or the combination of METTL3 overexpression, dimethyl sulfoxide (DMSO) was used as a vehicle for the negative control assays.

### RNA isolation and qRT‒PCR

Total RNA was isolated using RNeasy reagents TRIzol reagent (Invitrogen, USA), and qRT‒PCR was performed using an ABI 7500 instrument (Applied Biosystems, Foster City, CA, USA) with GoTaq® qPCR Master Mix (Promega, Wisconsin, USA) according to the manufacturer’s instructions. The primer sequences are provided in Additional file 1: Table S3.

### Western blot analysis

Cells were extracted by using RIPA lysis buffer containing protease inhibitors (R0010, Solarbio, China), and equal amounts of proteins were separated, and then transferred onto PVDF (0.45 μm) membranes (IPVH00010, Immobilon-P, Millipore). After being blocked with non-fat milk, the membranes were probed with the primary antibodies (1:1000) at 4 °C overnight. The next day, the membrane was washed and incubated with secondary antibodies (SA00001-1 or SA00001-2, Proteintech) for 2 h at room temperature, followed by detection with the chemiluminescence Tanon-5200 (Tanon, Shanghai, China). Antibodies were listed as follows: METTL3 (ab195352, Abcam, Cambridge, UK); phospho-PI3K P85 (TA3242, Abmart, Shanghai, China); PI3K (T40115, Abmart, Shanghai, China); mTOR (T55306S, Abmart, Shanghai, China); Histone H3 (4620S, CST, USA); p-mTOR(5536T, CST, USA); Histone H3 acetyl K9 (ab10812, Abcam, Cambridge, UK); H3K4me3 (PTM-5019, PTM Bio Inc., Hangzhou, China); H3K36me3 (PTM-625RM, PTM Bio Inc., Hangzhou, China); H3K27me3 (ABE44, Merck Millipore, USA). Other antibodies (GAPDH, VGF, phospho-AKT, AKT, KI67) were obtained from Proteintech Biotechnology (Wuhan, China). The original pictures of western blots are listed in Additional file 3.

### Cell proliferation assay

Based on the CCK-8 assay and 5-ethynyl-2’-deoxyuridine (EdU) incorporation assay, cell proliferation was quantified. For the CCK-8 assay, the transfected A549 cells were seeded on 96-well plates (1 × 10^3^/well) with 10 μl of CCK-8 reagent (FC101, TransGen, Beijing, China) and counted every 24 h for three days. Counting was carried out after cell attachment for 24 h (labeled 24 h). For the EdU incorporation assay, cell viability was estimated using EdU reagent (C0075s, Beyotime, China) following the manufacturer’s instructions. Briefly, the transfected A549 cells were incubated with EdU reagent for 2 h and processed. Then, the cells were fixed with 4% paraformaldehyde, fluorescent dye was used to stain cells, and the cells were visualized under a fluorescence microscope (Nikon laser confocal microscope, Nikon, Japan).

### Colony formation, cell invasion, and migration

For the colony formation assay, the transfected cells were seeded into 6-well plates (500/well) and maintained in DMEM containing 10% FBS for approximately 14 days. Colonies were fixed with 4% formaldehyde, stained with 0.1% crystal violet for 15 min, and statistically analyzed after being imaged and counted. For analysis of cell invasion and migration, 1 × 10^5^ cells were seeded in transwell plates coated or uncoated with Matrigel (356234, Corning, Acton, Massachusetts, USA). Following 24 h of incubation, cells were fixed in 4% paraformaldehyde for 30 min and stained with 0.1% crystal violet. Migrating and invading cells were observed with a light microscope (Nikon, Tokyo, Japan). In addition, for the scratch assay, A549 cells were seeded into 6‐well plates (2 × 10^5^/well), and when the cells reached full confluence, an artificial scratch was made with 10 µl sterile pipette tips. Then, cell migration ability was evaluated by the percentage of wound-healing rate (distance migrated/original wound distance × 100%) at 24 h and 48 h after wounding.

### MeRIP-qPCR assay

According to the manufacturer’s protocol, the MeRIP assay was conducted using the MeRIP Kit (P-9018, EPIGENTEK, Farmingdale, NY, USA). In brief, total RNA was randomly fragmented into a size of 100–200 nucleotides, and then RNA was immunoprecipitated with m6A antibody for further qRT‒PCR analysis. Based on the SRAMP website (http://www.cuilab.cn/sramp), specific primers were designed for qRT‒PCR analysis. The sequences of the primers are provided in Additional file 1: Table S4.

### Dual-luciferase reporter assay

VGF-CDS and SETD2-3'UTR fragments containing wild-type and mutant m6A motifs, were directly synthesized by Beyotime (Shanghai, China) and then inserted into the luciferase reporter vector (pmirGLO, Promega, Wisconsin, USA). Cells were seeded into each well of a 6-well plate and co-transfected with vectors according to the JetPRIME reagent protocol. After 48 h, according to the manufacturer's instructions, luciferase activity was determined using the Dual-Luciferase Reporter Assay System (Promega, Wisconsin, USA).

### ChIP assays

The ChIP assays were performed using the Simple ChIP Enzymatic Chromatin IP Kits (9003S, Cell Signaling Technology, USA) according to the manufacturer’s instructions. Quantification of immunoprecipitated DNA was analyzed using qRT‒PCR with SYBR Green Mix (Promega, Wisconsin, USA). The VGF promoter sequences of the primers are provided in Additional file 1: Table S5.

### In vivo tumor formation assay

For the in vivo tumor formation assay, transfected A549 cells (1 × 10^7^) were injected subcutaneously into the right thigh of female BALBc nude mice. One month after transplantation, the mice were sacrificed and the tissue samples were measured, weighed, and immunohistochemically analyzed. The tumor volume was calculated by the formula 1/2 × length × width^2^. Female BALB/c nude mice were purchased from Beijing SPF (Beijing, China) and were grouped randomly (5 or 6 mice per group). All mice were reared at the Animal Center of Inner Mongolia University, and all experiments were approved by the Animal Care and Use Committee of Inner Mongolia University (approval ID: IMU-mouse-2022-052).

### RNA stability

After gene manipulation, cells were treated with 10 μg/mL actinomycin D (SBR00013, Sigma-Aldrich, St. Louis, MI, USA) in the cell medium. Cells were harvested at 0, 2, 4, 6, and 8 h for RNA extraction. The levels of SETD2 mRNA levels were measured using qRT‒PCR the primers provided in Additional file 1: Table S3.

### Statistical analysis

Data were analyzed using Prism Graphpad 7.0 software (GraphPad Software, La Jolla, United States) and are presented as the mean ± standard deviation. Each experiment was replicated three times unless otherwise indicated. Significant differences were assessed by two‐tailed Student's t‐test or one-way ANOVA for comparisons between multiple groups. *: *p* value < 0.05, **: *p* value < 0.01. ***: *p* value < 0.001.

### Supplementary Information

**Additional file 1. Table S1**. Prognosis-associated genes. **Table S2**. The sequences of siRNAs. **Table S3**. Primer sequences of genes. **Table S4**. Primer sequences for MeRIP-qPCR. **Table S5**. Primer sequences of the VGF promoter.

**Additional file 2. Fig S1**. A–B CCK8 (A) and EdU (B) assays were used to assess cell viability and proliferation. C Colony formation assays. D The scratch assay experiments on A549 cells. E–F Cell migratory (E) and invasive (F) abilities were detected using transwell assays in A549 cells. G Forest map illustrates the survival analysis of m6A readers in LUAD. Bar = mean ± SD. *p  <  0.05, **p  <  0.01, ***p  <  0.001.

**Additional file 3**. Western blot raw data.

## Data Availability

The datasets used and analyzed during the current study are available from the corresponding author upon reasonable request.

## Abbreviations

METTL3: Methyltransferase 3
NSCLC: Non-small cell lung cancer
VGF: Nerve growth factor inducible
LUAD: Lung adenocarcinoma
MeRIP-seq: Methylated RNA immunoprecipitation sequencing
WGCNA: Weighted correlation network analysis
m6A: N6-methyladenosine
GO: Gene ontology
KEGG: Kyoto Encyclopedia of Genes and Genomes
qRT‒PCR: Quantitative real-time polymerase chain reaction
CDS: Coding sequences
TSS: Transcription start site
