# Correction: METTL3 exerts synergistic effects on m6A methylation and histone modification to regulate the function of VGF in lung adenocarcinoma

**DOI:** 10.1186/s13148-023-01607-5

**Published:** 2024-01-02

**Authors:** Kesong Shi, Rula Sa, Le Dou, Yuan Wu, Zhiqiang Dong, Xinyao Fu, Haiquan Yu

**Affiliations:** State Key Laboratory of Reproductive Regulation a Breeding of Grassland Livestock, School of Life Sciences, Inner Mongolia University, Hohhot, 010070 Inner Mongolia China

**Correction : Clinical Epigenetics (2023) 15:153** 10.1186/s13148-023-01568-9

Following publication of the original article [1], the authors noticed the errors in the figure. In Fig. 6D (si-NC 24 h) and Fig. 6D (si-METTL3-2 48 h) are identical, which was an inadvertent mistake by the author. The authors regret for the error and provided revised Fig. 6. This error has been corrected with this erratum which do not affect the results or conclusions.

**Fig. 6:**
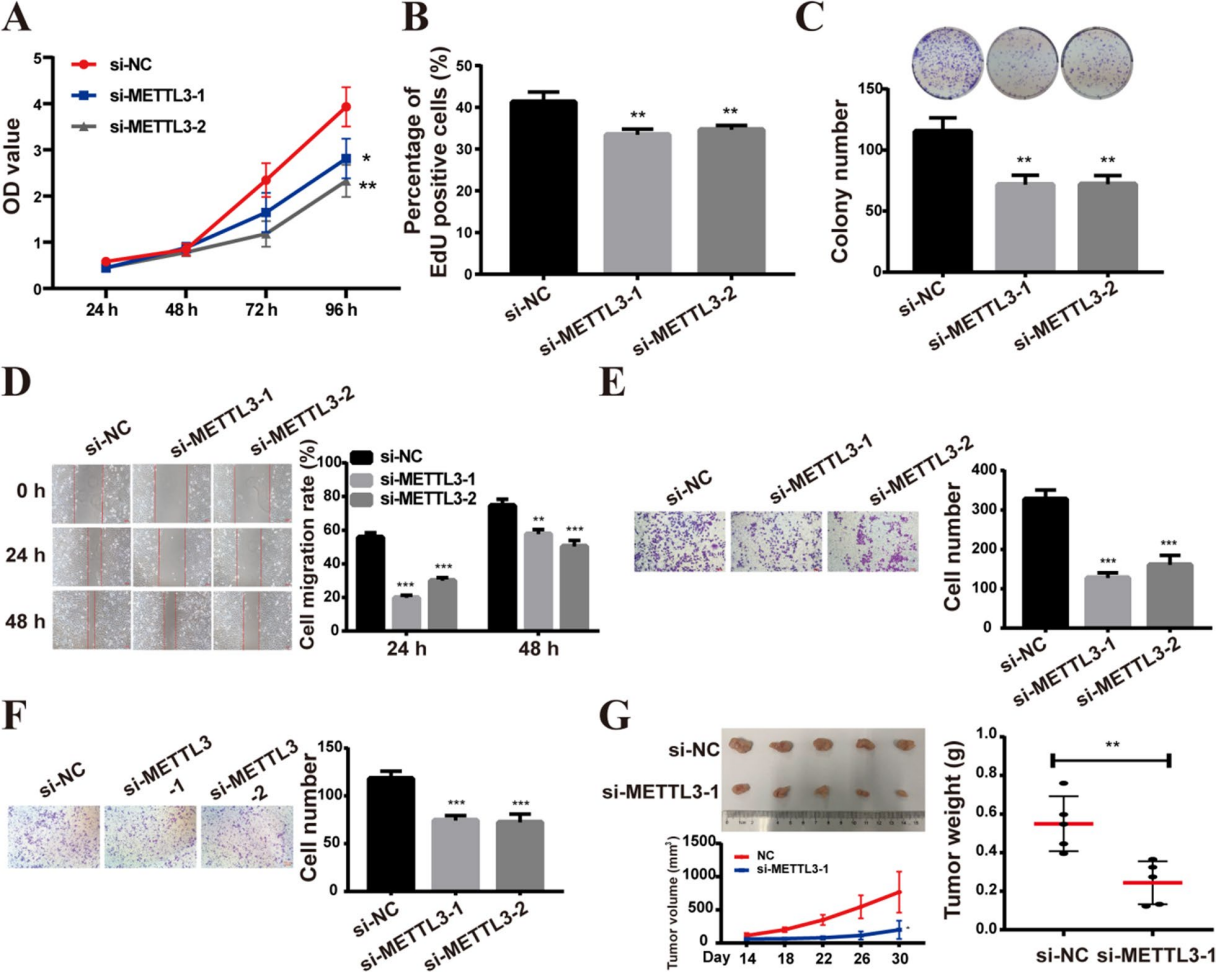
METTL3 knockdown inhibited the malignant phenotype of A549 cells in vitro. **A**, **B** CCK8 (**A**) and EdU (**B**) assays were used to assess cell viability and proliferation. **C** Colony formation assays. **D** The scratch assay experiments on A549 cells. **E**, **F** Cell migratory and invasive abilities were detected using transwell assays in A549 cells. **G** METTL3 knockdown inhibited the growth of subcutaneous xenografts in vivo (top left), the tumor growth curve (low left), and the weight of tumors xenografted in nude mice (right). Bar = mean ± SD. *p < 0.05, *p < 0.01, ***p < 0.001
