# Supplementary material for: METTL3 exerts synergistic effects on m6A methylation and histone modification to regulate the function of VGF in lung adenocarcinoma
Source: Clin Epigenetics. 2023 Sep 23;15:153. doi: 10.1186/s13148-023-01568-9 (PMC10517543; doi:10.1186/s13148-023-01568-9)
Supplement: Supplementary file 1 — Additional file 1. Table S1. Prognosis-associated genes. Table S2. The sequences of siRNAs. Table S3. Primer sequences of genes. Table S4. Primer sequences for MeRIP-qPCR. Table S5. Primer sequences of the VGF promoter. [file 13148_2023_1568_MOESM1_ESM.docx]

**Table S1. Prognosis-associated genes**

| Genes | Logrank *p* | HR | Genes | Logrank *p* | HR |
| --- | --- | --- | --- | --- | --- |
| ADAM12* | 0.0038 | 1.69(1.18-2.43) | ITIH5 | 0.0013 | 0.57(0.41-0.81) |
| TMEM184A | 0.0051 | 1.53(1.13-2.06) | ITLN1 | 0.025 | 0.7(0.51-0.96) |
| TEKT1 | 0.15 | 0.81(0.6-1.08) | VEPH1 | 0.15 | 0.8(0.59-1.08) |
| RASGRF1 | 0.0018 | 0.62(0.46-0.84) | AKR1B10 | 0.05 | 1.37(1-1.88) |
| ENTPD8 | 0.093 | 0.77(0.56-1.05) | CDC25C | 4.00E-05 | 1.9(1.39-2.59) |
| LRRK2 | 0.0061 | 0.67(0.5-0.89) | GRIK4 | 0.022 | 0.7(0.51-0.95) |
| HTR3A | 0.0015 | 1.63(1.2-2.22) | IL31RA | 0.26 | 1.19(0.88-1.62) |
| ECRG4 | 0.00052 | 0.52(0.36-0.76) | ABCA8 | 0.00045 | 0.51(0.34-0.75) |
| PAEP | 0.21 | 0.8(0.57-1.14) | C7 | 8.80E-05 | 0.45(0.3-0.68) |
| TMPRSS4 | 0.074 | 0.77(0.57-1.03) | C20orf202 | 0.054 | 1.36(0.99-1.85) |
| SPAG6 | 0.28 | 0.84(0.62-1.15) | SEMA6A | 0.38 | 1.14(0.85-1.53) |
| C1QTNF7 | 0.003 | 0.64(0.48-0.86) | HMGA2 | 0.0011 | 1.64(1.22-2.21) |
| SGO1 | 0.00031 | 1.73(1.28-2.34) | RXRG | 0.0042 | 0.57(0.38-0.84) |
| PGLYRP4 | 0.039 | 0.74(0.55-0.99) | PACRG | 0.17 | 1.23(0.91-1.66) |
| GNGT1 | 0.033 | 1.37(1.02-1.84) | TBX4 | 0.0025 | 0.59(0.42-0.83) |
| RHBDL1 | 0.34 | 1.16(0.86-1.56) | RETN | 0.0072 | 0.66(0.48-0.89) |
| PTPRQ | 0.00014 | 0.57(0.42-0.76) | CLPSL1 | 0.082 | 1.3(0.97-1.74) |
| ARHGEF15 | 0.023 | 0.7(0.51-0.95) | LDB2 | 0.015 | 0.67(0.48-0.93) |
| SEMA6D | 0.00074 | 0.61(0.46-0.82) | BUB1B | 4.00E-05 | 1.92(1.4-2.63) |
| NTNG1 | 0.098 | 0.78(0.59-1.05) | FAM189A2 | 0.00043 | 0.58(0.42-0.79) |
| DLGAP5 | 3.00E-05 | 1.99(1.43-2.77) | SALL4 | 0.18 | 1.22(0.91-1.64) |
| SH2D4B | 0.049 | 0.75(0.56-1) | GLB1L3 | 0.0014 | 0.62(0.47-0.84) |
| C12orf56 | 0.11 | 1.27(0.95-1.7) | RADIL | 0.17 | 0.81(0.59-1.1) |
| DLC1 | 0.002 | 0.56(0.38-0.81) | EFNA3 | 0.0048 | 1.56(1.14-2.12) |
| VGF | 1.00E-04 | 1.77(1.32-2.37) | SIGLEC11 | 0.013 | 0.69(0.51-0.93) |
| SLIT2 | 0.066 | 0.74(0.53-1.02) | MMRN1 | 0.028 | 0.67(0.47-0.96) |
| PCOLCE2 | 0.22 | 1.2(0.9-1.62) | ONECUT2 | 0.15 | 1.24(0.93-1.67) |
| IGF2BP1 | 1.40E-06 | 2.08(1.53-2.82) | RNF183 | 0.15 | 0.8(0.59-1.09) |
| HELT | 0.051 | 0.7(0.49-1) |  |  |  |

* The red background indicates a significant correlation with the prognosis **(**Logrank *p* < 0.01).

**Table S2. The sequences of siRNAs**

| Target | Sequences |
| --- | --- |
| siRNA-control | S: UUCUCCGAACGUGUCACGUTT  AS: ACGUGACACGUUCGGAGAATT |
| siRNA-METTL3-1 | S: GCUGCACUUCAGACGAAUUTT  AS: AAUUCGUCUGAAGUGCAGCTT |
| siRN -METTL3-2 | S: CCUGCAAGUAUGUUCACUATT  AS: UAGUGAACAUACUUGCAGGTT |
| siRNA-VGF-1 | S: UCUGCUGAUCAACGGGAAUTT  AS: UAACCCGUUGAUCAGCAGATT |
| siRNA-VGF-2 | S: UCAUUGAGCUGUCCACCAATT  AS: UUGGUGGACAGCUCAAUGATT |

**Table S3. Primer sequences of genes**

| Genes | Sequences (5’-3’) |
| --- | --- |
| GAPDH | F: 5’- GCACCGTCAAGGCTGAGAAC -3’ |
|  | R:5’- TGGTGAAGACGCCAGTGGA -3’ |
| VGF | F: 5’- GGAACTGCGAGATTTCAGTCC -3’ |
|  | R: 5’- GTGCGGGTTTCCGTCTCTG -3’ |
| METTL3 | F: 5’-CAAGCTGCACTTCAGACGAA-3’ |
|  | R: 5’-GCTTGGCGTGTGGTCTTT-3’ |
| TBX4 | F: 5’-TGGAAGAAGTTCCACGAGGC-3’ |
|  | R: 5’-CTTCCCTGCCACCATCCATT-3’ |
| C1QTNF7 | F: 5’-ATCTGCAGCATTCCTGGCTT-3’ |
|  | R: 5’-CTTTCTCACCGGCAAGACCT-3’ |
| HMGA2 | F: 5’-AGCCCTCTCCTAAGAGACCC-3’ |
|  | R: 5’-GCAAGGCAACATTGACCTGAG-3’ |
| BUB1B | F: 5’-CAGGTCTTCTGGGATGGGTC-3’ |
|  | R: 5’-CATTTCTGCTGCAAGCTCCC-3’ |
| SETD2 | F: 5’-CCGACCCCTGAAGAAGAAGAA-3’ |
|  | F: 5’-GACACCTTCTGTCGTCCCTG-3’ |

**Table S4. Primer sequences of MeRIP-qPCR**

| Genes | Sequences |
| --- | --- |
| VGF | F: 5’- GAGGAGGTGGAGGAGAAGCG -3’  R: 5’- GGATGTAGTTGGGGAAAGGG -3’ |
| 1# SETD2 | F: 5’- TACACAGATAACAGAGCACG-3’  R: 5’- TCATTAGGGGGAGAACAACA -3’ |
| 2# SETD2 | F: 5’- TAGTAGGTGCAAAGAAAAAG-3’  R: 5’- ATAACTGGCATAGACATGAG-3’ |
| 3# SETD2 | F: 5’- TAGATGCCACTCAAAAAGGA-3’  R: 5’- CTGGAACTGATAGTCAAACG-3’ |
| 4# SETD2 | F: 5’- ATCGCGTGCTCATACACCAC-3’  R: 5’- CTTTGCCATCCTTGCCTTCT-3’ |
| 5# SETD2 | F: 5’- CCTCCACCAGTACCAGTGGT-3’  R: 5’- TGTCTGTCCTTGATAATATA-3’ |
| 6# SETD2 | F: 5’-AGGATGGGTGGTCAGGTAAG-3’  R: 5’-GATGGAGTTCATTTTTGTGG-3’ |

**Table S5. The VGF promoter sequences of primers**

| Gene | Sequences |
| --- | --- |
| VGF | F: 5’-CCTCTCTCACTGGGGTTTCC-3’  R: 5’-TCTGTTTTCTCCCTCTGCCT-3’ |
