# Supplementary figures and images for: METTL3 exerts synergistic effects on m6A methylation and histone modification to regulate the function of VGF in lung adenocarcinoma
Source: Clin Epigenetics. 2023 Sep 23;15:153. doi: 10.1186/s13148-023-01568-9 (PMC10517543; doi:10.1186/s13148-023-01568-9)

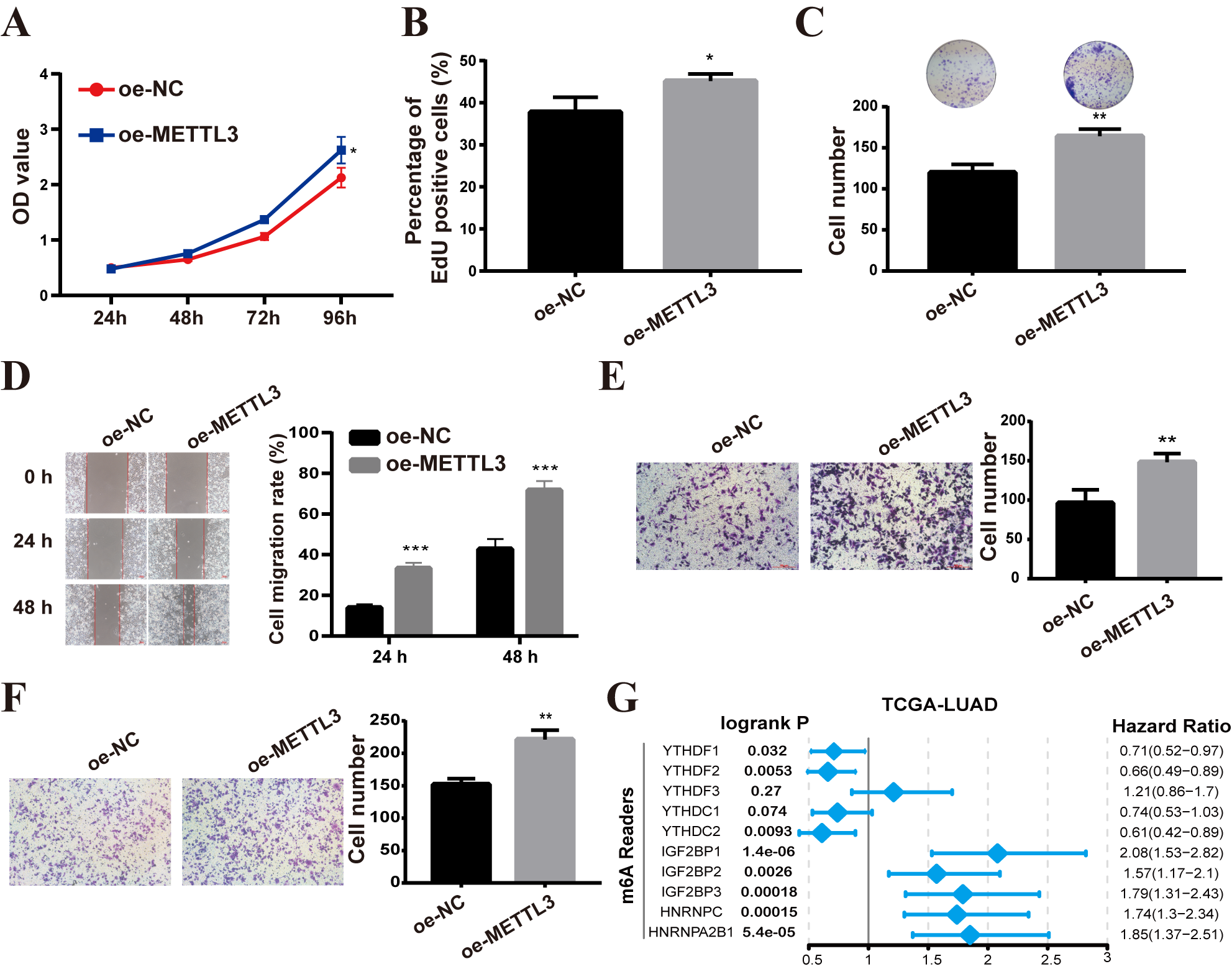

Supplement: Supplementary file 2 — Additional file 2. Fig S1. A–B CCK8 (A) and EdU (B) assays were used to assess cell viability and proliferation. C Colony formation assays. D The scratch assay experiments on A549 cells. E–F Cell migratory (E) and invasive (F) abilities were detected using transwell assays in A549 cells. G Forest map illustrates the survival analysis of m6A readers in LUAD. Bar = mean ± SD. *p < 0.05, **p < 0.01, ***p < 0.001. [file 13148_2023_1568_MOESM2_ESM.tif]

**Additional file 3**

**Western blot raw data**

**Fig. 2D**


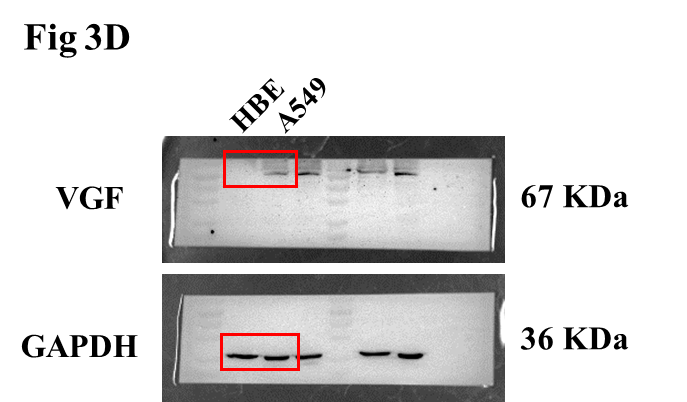


**Fig. 3B**


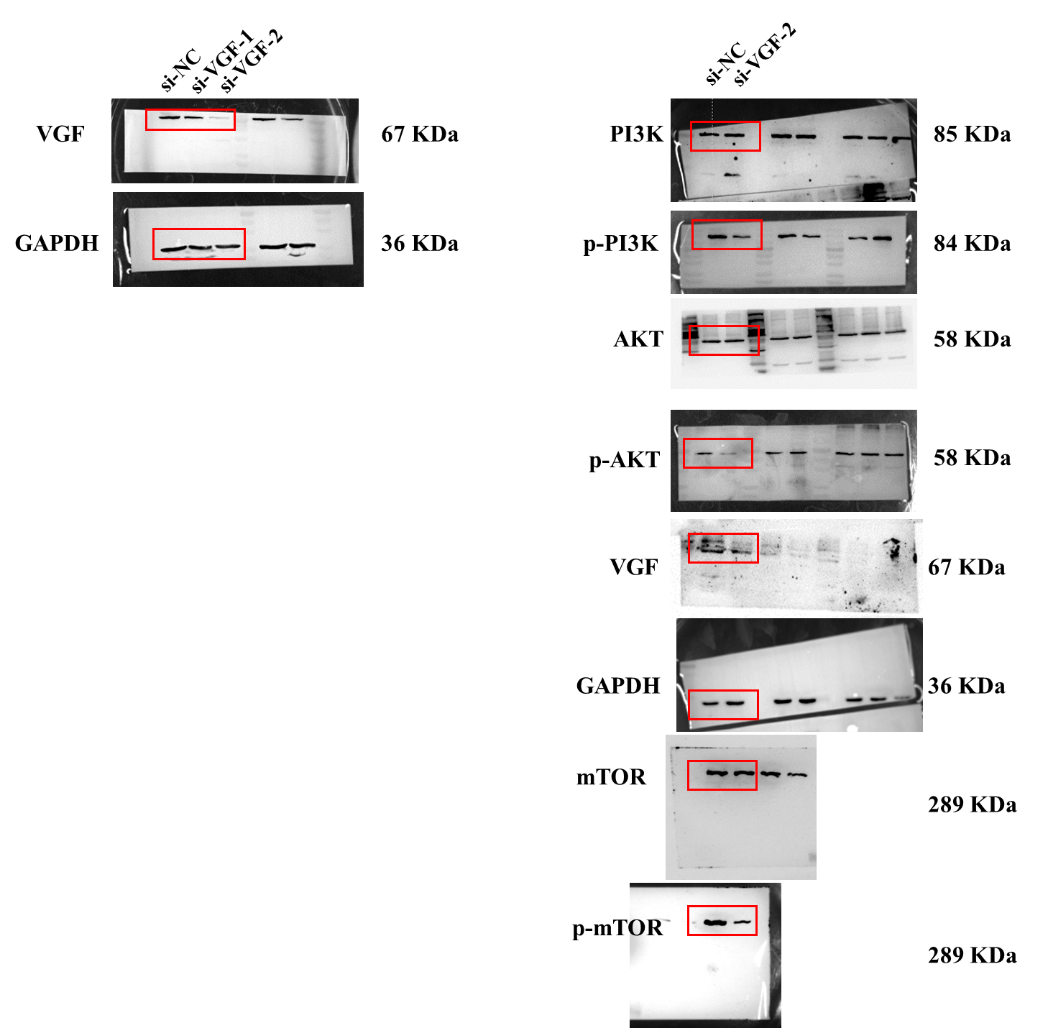


**Fig. 5C**


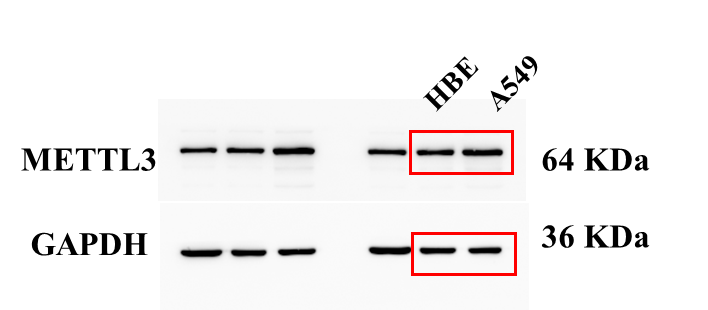


**Fig. 5H**


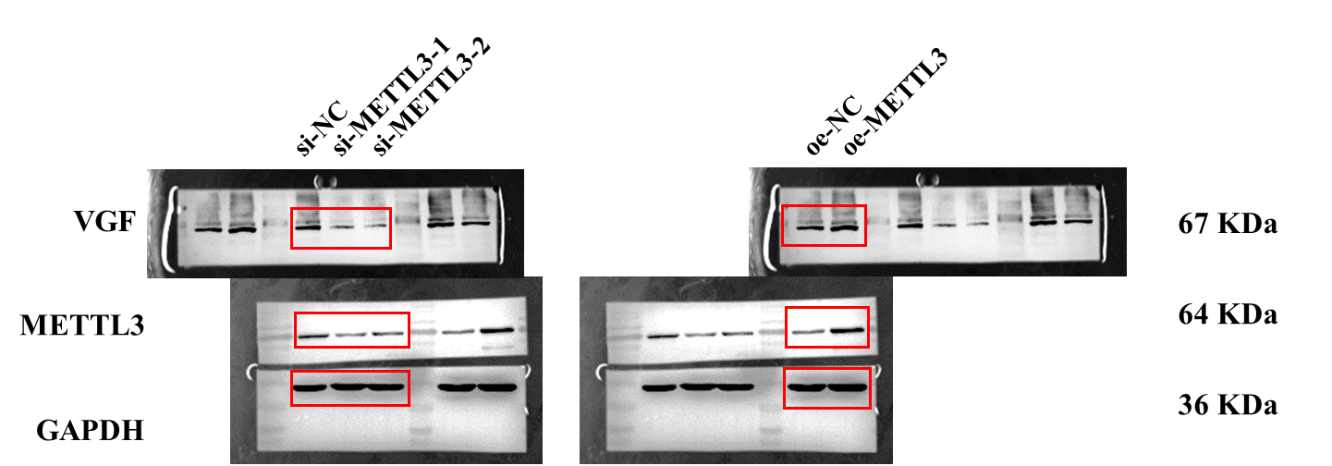


**Fig. 7J**


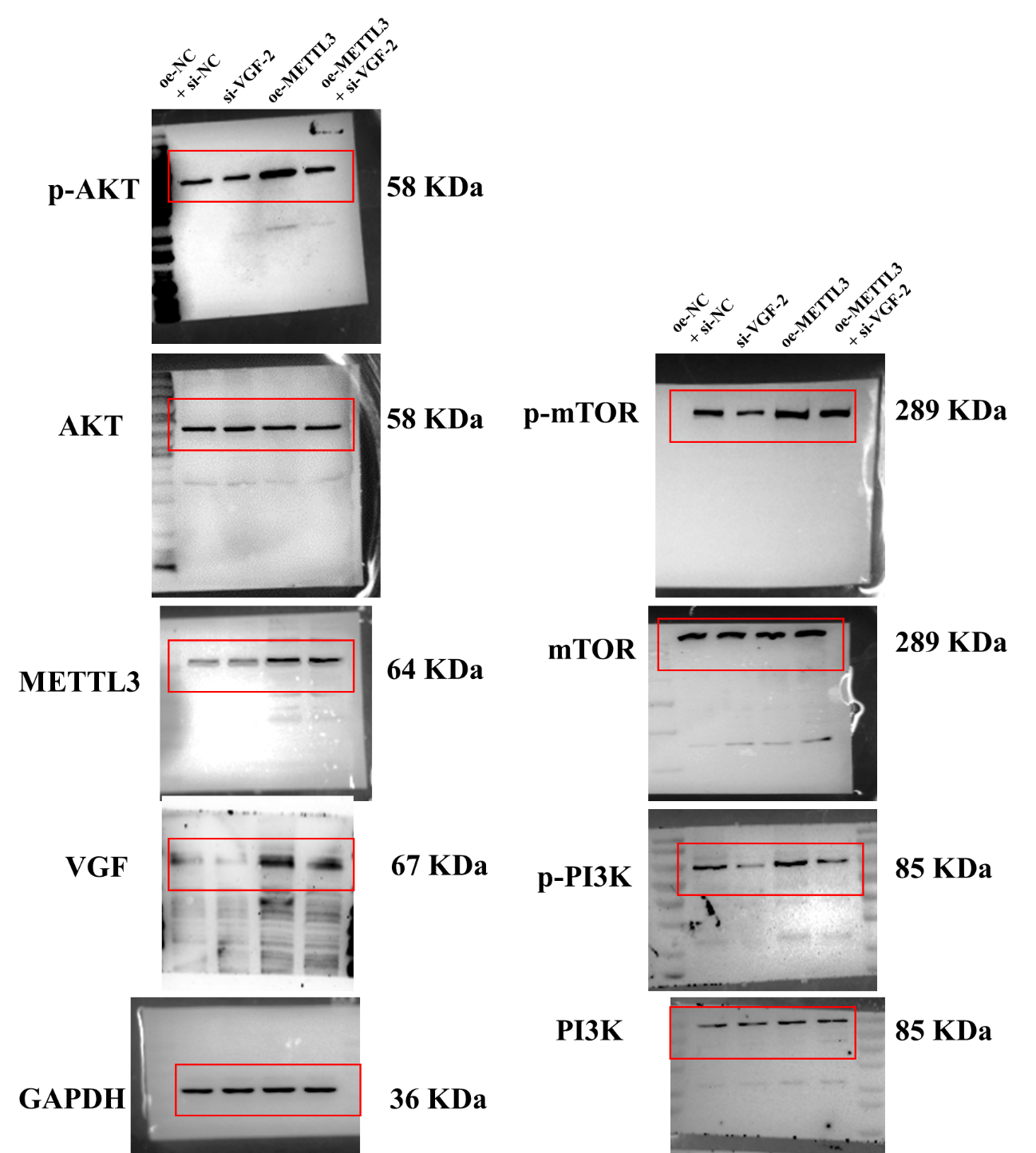


**Fig. 7K**


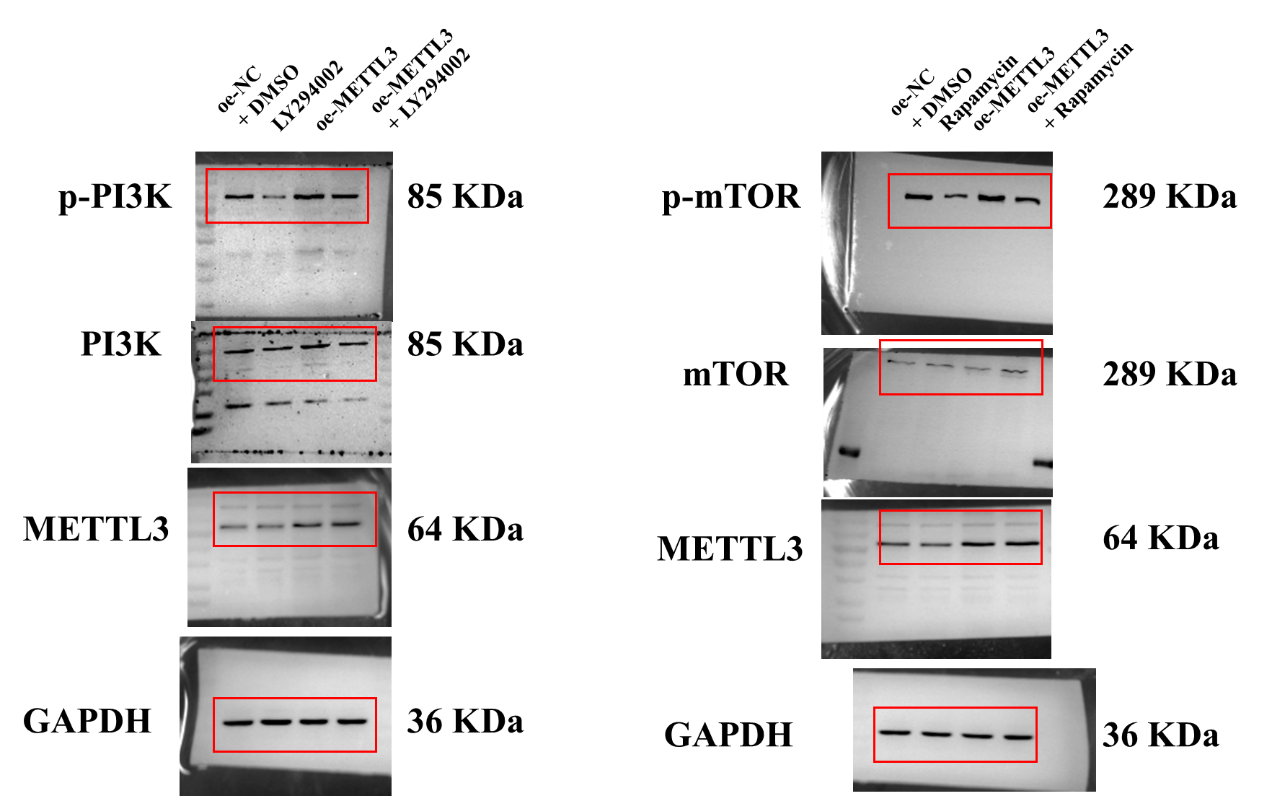


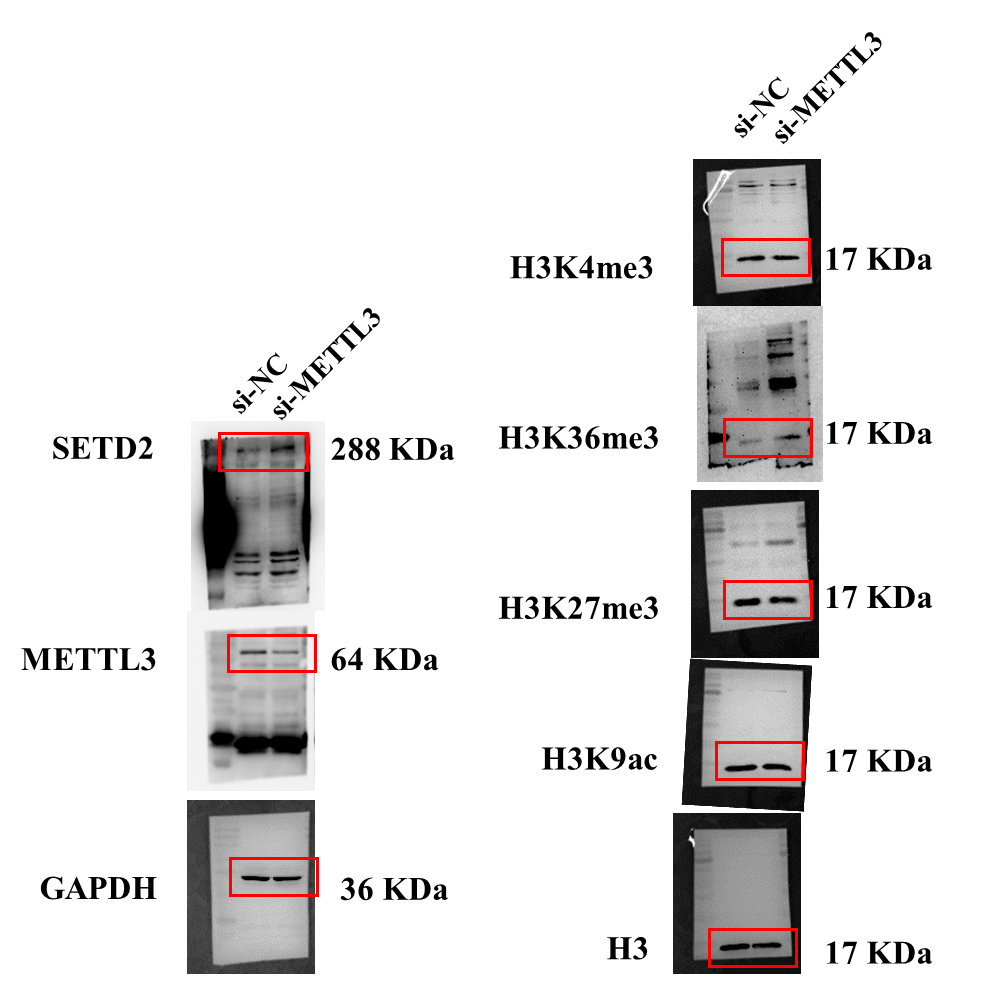


**Fig. 7L**

Supplement: Supplementary file 3 — Additional file 3. Western blot raw data. [file 13148_2023_1568_MOESM3_ESM.docx]
